# High‐Performance, Roll‐to‐Roll Fabricated Scaffold‐Supported Solid Electrolyte Separator for Practical All‐Solid‐State Batteries

**DOI:** 10.1002/smll.202502996

**Published:** 2025-07-01

**Authors:** Seok Hun Kang, Hyobin Lee, Young‐Jin Hong, Seokhan Myoung, Hyewon Seo, Jaecheol Choi, Seokyoon Yoon, Ju Young Kim, Dong Ok Shin, Myeong Ju Lee, Young‐Sam Park, Young‐Gi Lee, Yong Min Lee

**Affiliations:** ^1^ Materials and Components Research Division Electronics and Telecommunications Research Institute (ETRI) 218 Gajeongno, Yuseong‐gu Daejeon 34129 Republic of Korea; ^2^ Department of Energy Science and Engineering Daegu Gyeongbuk Institute of Science and Technology (DGIST) 333 Techno Jungang‐daero, Dalseong‐gun Daegu 42988 Republic of Korea; ^3^ R&D Center MinTech Co., Ltd 62 Gukjegwahak 21‐ro, Yuseong‐gu Daejeon 34002 Republic of Korea; ^4^ Department of Chemical and Biomolecular Engineering Yonsei University 50 Yonsei‐ro, Seodaemun‐gu Seoul 03722 Republic of Korea

**Keywords:** all‐solid‐state batteries, high energy density, laser‐drilled scaffold, roll‐to‐roll fabrication, solid electrolyte separator

## Abstract

All‐solid‐state batteries (ASBs) are promising candidates for next‐generation energy storage systems due to their enhanced safety and potential for higher energy densities. However, achieving practical ASBs with energy densities surpassing those of state‐of‐the‐art lithium‐ion batteries (LIBs) requires the development of thin, mechanically robust solid electrolyte separators (SESs). In this study, a scalable tape casting method is employed to fabricate a thin SES with a thickness of 27 µm and a high ionic conductance of 146 mS cm^−2^. The SES, composed of Li_6_PS_5_Cl SE and a laser‐drilled porous polyimide (PI) scaffold with a high porosity of 69%, exhibits a tensile stress of 7.15 MPa at 6% strain, demonstrating the mechanical integrity necessary for commercial roll‐to‐roll fabrication. Due to its reduced thickness, the LiNi_0.83_Co_0.11_Mn_0.06_O_2_||Li‐In pouch cell achieves outstanding estimated cell‐level gravimetric and volumetric energy densities of 322 Wh kg^−1^ and 571 Wh L^−1^, respectively, demonstrating its practical viability. Additionally, simulation studies highlight the importance of optimizing the porosity and pore distribution of porous scaffolds to minimize Li‐ion flux heterogeneity and prevent non‐uniform Li plating in scaffold‐supported SESs. Finally, a 4 m long, double‐side coated SES is successfully manufactured using an industrial‐level comma coater, demonstrating the feasibility of the approach for large‐scale SES production and the forthcoming commercialization of ASBs.

## Graphical abstract

A scalable scaffold‐supported solid electrolyte separator (SES) with a thickness of 27 µm and high ionic conductance is fabricated via laser drilling and tape casting. Mechanical and multiphysics simulations guide the design of the porous scaffold. The SES enables high cell‐level energy density of 322 Wh kg^−1^ and excellent mechanical durability, supporting roll‐to‐roll (R2R) processing for practical all‐solid‐state batteries.

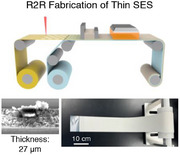

## 1 Introduction

The demand for higher energy density and enhanced safety in energy storage devices has driven the search for alternative technologies to conventional lithium‐ion batteries (LIBs). All‐solid‐state batteries (ASBs) have emerged as promising candidates due to their potential to mitigate the safety concerns associated with flammable liquid electrolytes, utilize high energy density lithium metal anodes (3860 mAh g^−1^), and enable compact bipolar stack configuration for enhanced energy density and reduced cost.^[^ ^1^, ^2^ ^]^ Among the various types of solid electrolytes (SEs) developed, sulfide‐based SEs are particularly promising for near‐future commercialization due to their high room temperature ionic conductivity and favorable mechanical ductility, which facilitate intimate solid–solid contact between active materials and SE particles through simple cold pressing.^[^ ^3^, ^4^ ^]^ However, the processing protocols of sulfide‐based ASBs present multiple technical challenges that must be addressed to harness the full potential of ASBs. The conventional approach of cold pressing SE powders into dense pellets using in‐house molds poses significant hurdles for mass production and achieving high cell‐level energy density due to the increased thickness and inherent brittleness of sulfide SE pellets.^[^ ^5^ ^]^

Recent research efforts have focused on developing sheet‐type solid electrolyte separators (SESs) to overcome these limitations, leading to significant improvements in SES thickness and ionic conductance. Whiteley et al. developed a freestanding sulfide‐polymer hybrid electrolyte membrane composed of Li_2_S‐P_2_S_5_ SE and polyimine, achieving a thickness of 64 µm and an ionic conductance of 7 mS cm^−2^.^[^ ^6^ ^]^ Oh et al. further enhanced the SES ionic transport by fabricating a freestanding SES using Li_6_PS_5_Cl SE, nitrile butadiene rubber (NBR) binder, and solvate ionic liquid, achieving an impressive ionic conductance of 471 mS cm^−2^ (calculated from ionic conductivity of 3.3 mS cm^−1^) with a thickness of 70 µm.^[^ ^7^ ^]^ Meanwhile, Wang et al. demonstrated an ultrathin freestanding SES composed of Li_6_PS_5_Cl SE and polytetrafluoroethylene (PTFE) binder, achieving a thickness of 20 µm and an ionic conductance of 667 mS, using a dry fabrication process.^[^ ^8^ ^]^

Despite these advancements, freestanding membranes composed solely of SE particles and binders (and, in some cases, small amounts of ionic liquid) suffer from poor mechanical integrity. The ionically insulating nature of the binder impedes ionic transport, and efforts to reduce the binder content to improve ionic conductance paradoxically lead to further reduction in mechanical robustness. This critical limitation impedes the integration of freestanding SE membranes into large‐scale manufacturing procedures such as roll‐to‐roll fabrication, which requires sufficient mechanical strength.

To address this issue, scaffold‐supported approaches have been proposed to enhance SES mechanical robustness.^[^ ^5^, ^9^ ^]^ While incorporating a scaffold inevitably increases overall SES thickness and ionic transport tortuosity, resulting in reduced ionic conductivity compared to freestanding membranes, scaffold‐supported systems provide significant mechanical advantages, making them more feasible for commercialization. For example, Nam et al. developed a thin SES by mechanically transferring a slurry‐processed Li_3_PS_4_ film onto a nonwoven poly(para phenylene terephthalamide) (PPTA) scaffold, achieving a 70 µm thick SE film with ionic conductivity of 0.20 mS cm^−1^.^[^ ^10^ ^]^ Although the concept was successfully demonstrated, the high thickness and complex fabrication process limited its commercial feasibility. Kim et al. reported a 40 µm thick SE membrane with an ionic conductivity of 0.058 mS cm^−1^ using a porous electrospun polyimide scaffold infiltrated with solution‐processed Li_6_PS_5_Cl_0.5_Br_0.5_.^[^ ^11^ ^]^ This approach simplified fabrication through scalable slurry casting, but ionic conductivity was reduced due to the use of polar ethanol as the solvent.

A key challenge in SE slurry fabrication is selecting an appropriate solvent and binder that are inert to highly reactive sulfide SEs. Despite extensive research to identify suitable solvents and binders for liquid‐phase sulfide SE fabrication, all reported dissolution‐based SE slurries have resulted in reduced ionic conductivity, typically in the 10^−5^–10^−4^ S cm^−1^ range.^[^ ^12^, ^13^, ^14^ ^]^ This reduction is expected due to the structural collapse and reconstruction of the argyrodite phase during the dissolution process. To preserve the high ionic conductivity of argyrodite SEs after liquid‐phase processing, suspension‐type SE slurries using nonpolar solvents unreactive to sulfide SEs are necessary.

Consequently, a robust scaffold with macropores ranging from tens to hundreds of micrometers is optimal for suspension‐type SE slurry coating, as it maintains the crystal structure of highly conductive argyrodite‐type sulfide SEs while allowing infiltration of micrometer‐sized SE particles. Our group previously demonstrated a thin and mechanically robust SES using a net‐type scaffold.^[^ ^15^ ^]^ Net or mesh‐type scaffolds satisfy the macropore requirements for suspension‐type slurry coating of argyrodite SEs, achieving a SE membrane with high ionic conductance of 84 mS cm^−2^ and a thickness of 66 µm. However, further reducing the interwoven net scaffold's thickness presents additional processing challenges.

In this study, we introduce a proof‐of‐concept fabrication strategy for thin and mechanically robust SESs through a scaffold‐supported approach applicable to various scaffold materials. Leveraging laser fabrication technology, macropores of desired dimensions can be fabricated on template films, achieving a porosity of up to 79% in our experimental demonstration. The versatility of laser fabrication enables fine‐tuning of the porous structure to optimize the balance between mechanical properties and scaffold porosity based on the selected scaffold material. Two distinct scaffold materials, polymer and metal, were employed to illustrate this adaptability. The resulting SES exhibits flexibility and excellent mechanical robustness, making it suitable for roll‐to‐roll manufacturing. Furthermore, our slurry‐casting approach enables industrial‐scale production, which we successfully demonstrate by fabricating a 100 mm wide, 4 m long, double‐side coated SES using an industrial‐level comma coater. This method aligns with existing LIB manufacturing infrastructure, offering substantial cost reductions and accelerating the commercialization of this technology. The electrochemical performance of the scaffold‐based SES was evaluated using symmetric Li‐In||Li‐In cells and LiNi_0.83_Co_0.11_Mn_0.06_O_2_(NCM)||Li‐In half cells. Finally, we demonstrate a practical ASB pouch cell with high estimated energy densities of 322 Wh kg^−1^ and 571 Wh L^−1^, calculated based on the total mass and volume of the cell, including the cathode, SES, anode, and current collectors.

## 2 Results and Discussion

In this study, the laser drilling method was employed as a scalable and versatile approach to fabricate porous scaffolds for thin and mechanically robust SESs. The focused high‐energy laser beam effectively cuts through a wide range of materials, from soft polymers to hard metals, demonstrating its adaptability to various template types. To showcase this versatility, two representative scaffold materials, polyimide (PI) and Ni foils, were selected. Among several polymer candidates (e.g. polyethylene terephthalate (PET), orientated polypropylene (OPP), and polyethylene naphthalate (PEN)), PI was chosen for its superior tensile strength (Figure S1a, Supporting Information), along with its excellent thermal stability, chemical resistance, and compatibility with battery operation, as reported in the literature.^[^ ^11^ ^]^ Ni was selected for its high tensile strength (Figure S1b, Supporting Information), chemical compatibility with sulfide SEs, and excellent manufacturability, with thicknesses as low as 3 µm achievable using state‐of‐the‐art technology.

**Figure 1:**
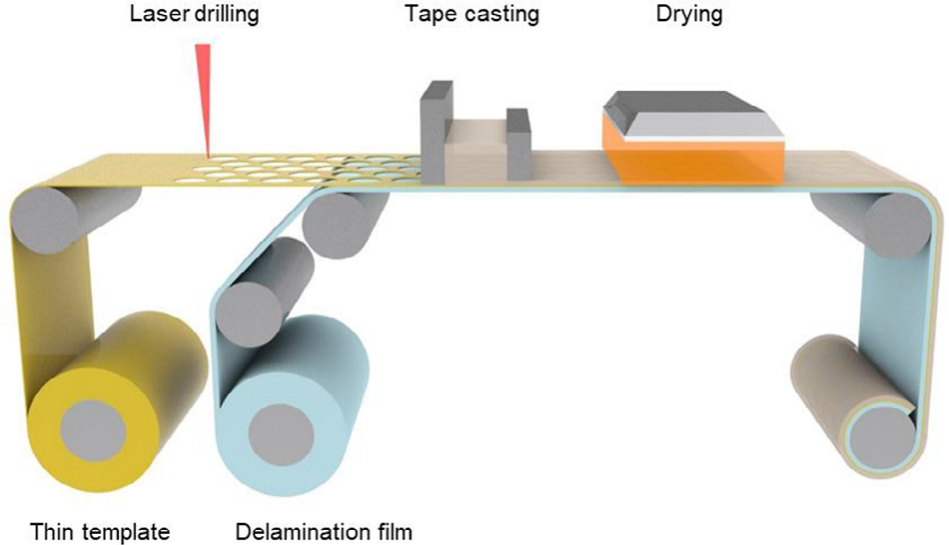
Schematic representation of roll‐to‐roll fabrication of SES using laser‐drilled scaffold.

**Figure** **1** illustrates the schematic process for roll‐to‐roll fabrication of SESs using laser‐drilled scaffolds. The microscale precision of the laser beam enables the creation of holes ranging from micrometers to centimeters scale. In this study, hole dimensions of a few hundred micrometers were identified as optimal, striking a balance between mechanical strength and porosity. Holes smaller than 100 µm reduce scaffold porosity, hinder SE slurry infiltration, and significantly increase processing time, posing scalability challenges. By adjusting hole diameter, gap distance, and array patterns, the overall punched area can be precisely controlled, as summarized in Table S1 (Supporting Information).

Increasing the punched area of the scaffold reduces the ionic tortuosity of the SES after tape casting the SE slurry onto the porous scaffold, thereby enhancing ionic conductivity. In this study, a punched area of up to 78% was achieved using a hole diameter of 400 µm, a gap distance of 30 µm, and a triangular array pattern. This level of porosity surpasses that of commercial separators (typically ≤ 50%) and, more importantly, features macropores of several hundred micrometers that facilitate the infiltration of suspension‐based SE slurry during SES fabrication.^[^ ^15^, ^16^ ^]^

**Figure 2:**
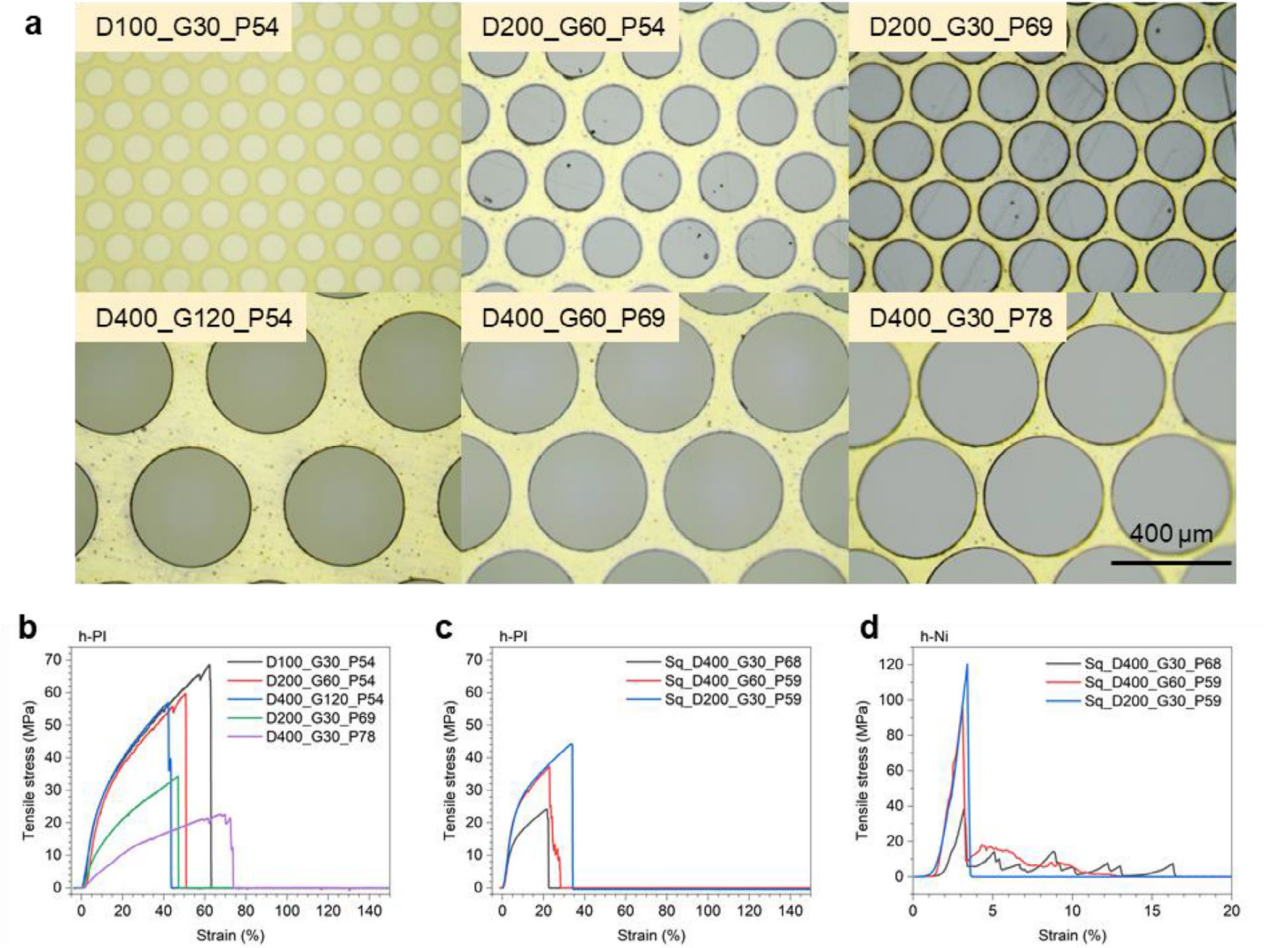
a) OM images of laser‐drilled h‐PI film, with hole diameter X µm, gap distance Y µm, corresponding punched area Z%. Tensile stress–strain curves of b,c) h‐PI and d) h‐Ni scaffolds.

In addition to porosity, the effects of pore uniformity and shape on SES performance were also investigated. Ionic transport simulations showed that scaffolds with more uniformly distributed holes exhibited enhanced ionic conductivity, as a result of reduced tortuosity and more efficient ion flux pathways (Figure S2, Supporting Information). Tensile simulations further revealed that scaffolds with round‐shaped holes generated lower stress concentrations than those with square holes under the same applied strain (Figure S3, Supporting Information), confirming that pore geometry also plays a key role in mechanical durability. Based on these findings, all scaffolds in this study were fabricated with round, uniformly distributed holes to optimize both mechanical and electrochemical performance. The precision of the laser‐drilled holes is illustrated in the optical microscopy (OM) images in **Figure** **2**a, Figures S4 and S5 (Supporting Information). Various hole arrays, denoted as DX_GY_PZ (hole diameter X µm, gap distance Y µm, and corresponding punched area Z%), exhibit cleanly cut spherical holes with minimal deviations at the edges. Both PI and Ni templates were accurately drilled, resulting in well‐defined dimensions.

In addition to high porosity, mechanical robustness is critical for integration into large‐scale SES production. Commercial roll‐to‐roll processes for LIBs require a minimum web tension of 20–70 N. Furthermore, SES should ultimately develop toward a thickness comparable to commercial LIB separators, which can be as thin as 15 µm. Based on these parameters, the required tensile strength for SESs in industrial comma coaters is estimated to range from 2.2 to 15.6 MPa, as detailed in Table S2 (Supporting Information).

To optimize the scaffold design for both mechanical strength and ionic conductivity, tensile tests were conducted on various laser‐drilled structures. The tensile stress–strain curves for laser‐drilled holey‐PI (h‐PI) and holey‐Ni (h‐Ni) scaffolds are shown in Figure 2b–d. As expected, increasing scaffold porosity compromises its tensile strength. However, tensile strength can be optimized by decreasing the hole diameter and gap distance. Additionally, adopting a triangular hole array pattern was found to enhance tensile strength, even at higher porosities. This was further validated by tensile simulations conducted under a constant force, where the triangular array exhibited lower deformation compared to a square array, indicating greater stiffness and improved mechanical robustness (Figure S6, Supporting Information). Among the tested structures, the h‐PI scaffold with the D200_G30_P69 structure exhibited a tensile strength of 34.0 MPa, sufficient for industrial comma coater applications, while maintaining a high porosity of 69%. Therefore, this design was selected as the optimal scaffold structure for SES fabrication.

To further demonstrate the versatility of the laser drilling technique, h‐Ni scaffolds were also fabricated. Owing to the superior mechanical properties of metal, h‐Ni scaffolds exhibited significantly higher tensile strength than h‐PI scaffolds. For example, the h‐Ni scaffold with the Sq_D200_G30_P59 structure exhibited a tensile strength of 120.3 MPa, while the h‐PI scaffold with the same structure displayed a tensile strength of 44.7 MPa. The inclusion of Ni scaffolds in this study highlights their potential in applications where ultrathin, high‐porosity SESs are required but polymeric scaffolds may lack adequate mechanical support, particularly at thicknesses below 5 µm. In such cases, metal‐based scaffolds could serve as mechanically robust alternatives suitable for advanced roll‐to‐roll processing or integration into multilayered ASB architectures.

**Figure 3:**
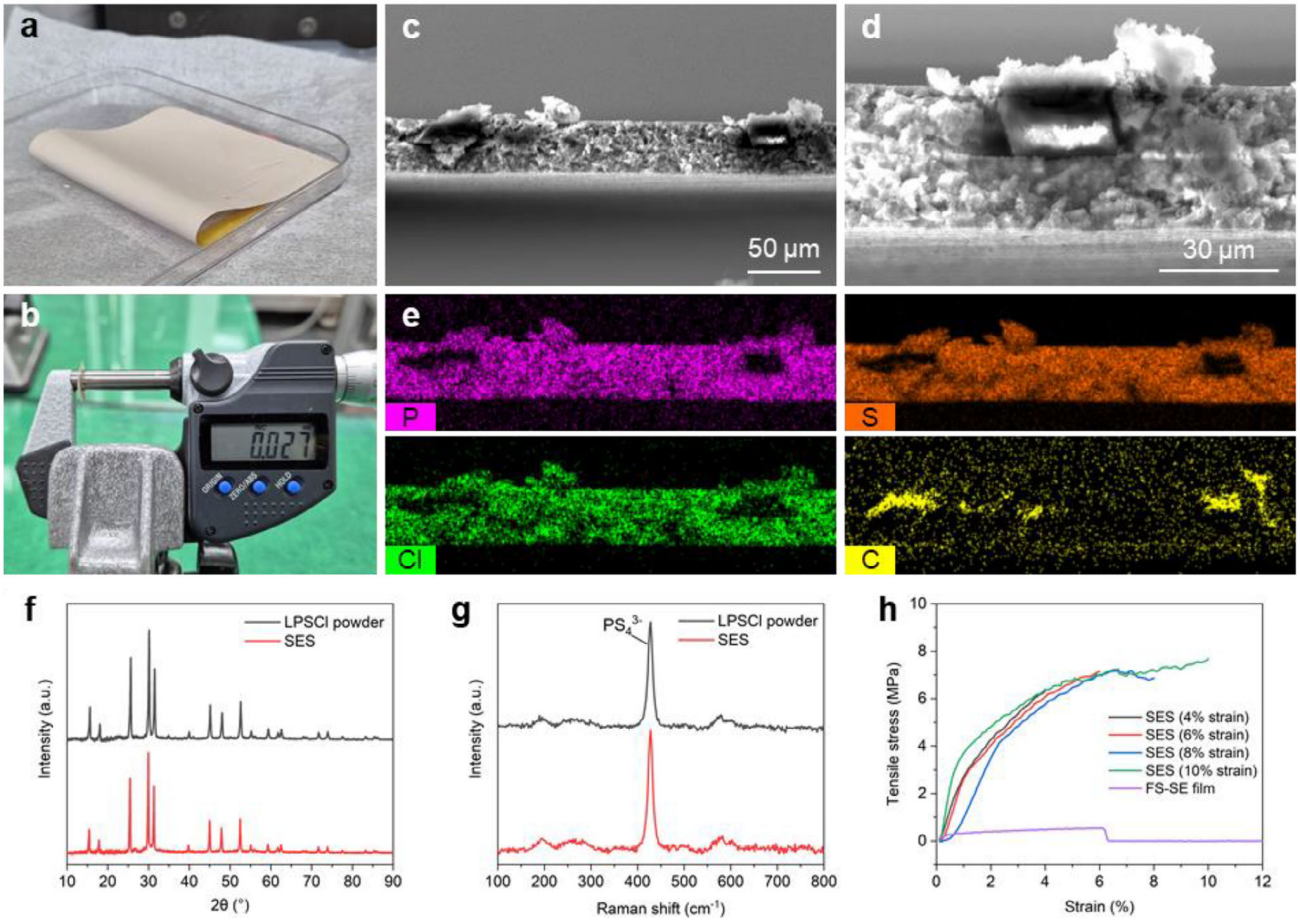
a) Photograph of the fabricated SES and b) its thickness measurement. Cross‐sectional SEM images at c) lower and d) higher magnification. e) EDS mapping images of (c). f) XRD and g) Raman spectra of LPSCl powder and SES. h) Tensile stress–strain curves of SES and FS‐SE film tested at various target strains.

By utilizing an 8 µm‐thick h‐PI scaffold, thin SESs were fabricated via a simple and scalable tape casting method. A photograph image of the fabricated SES (**Figure** **3**a) demonstrates its high flexibility, attributed to the flexible PI scaffold and the nitrile butadiene rubber (NBR) binder that securely holds the SE matrix together. The thinnest double‐side coated SES fabricated in this study demonstrated a total thickness of 27 µm (Figure 3b). Cross‐sectional scanning electron microscopy (SEM) and energy‐dispersive X‐ray spectroscopy (EDS) images (Figure 3c–e), reveal a dense and robust SE matrix enveloping the h‐PI strips. The high porosity of 69% minimizes ionic tortuosity from the scaffold, facilitating efficient ionic transport. EDS mapping confirms the uniform dispersion of P, S, and Cl signals, corresponding to the Li_6_PS_5_Cl (LPSCl) SE, and the C signal, corresponding to the PI scaffold.

To confirm the retention of the argyrodite crystal structure of LPSCl after liquid‐phase processing, X‐ray diffraction (XRD) analysis was conducted on both pristine SE powder and the fabricated SES. As shown in Figure 3f, the characteristic peaks of the argyrodite phase are well‐maintained in the SES, suggesting that the high ionic conductivity of LPSCl is preserved.^[^ ^17^ ^]^ The absence of impurity peaks further indicates excellent compatibility of LPSCl with NBR and anisole. Raman spectroscopy corroborates these findings, as the spectra exhibit a distinct peak near 428 cm^−1^, corresponding to PS_4_^3−^, for both the pristine SE and the SES, confirming the structural retention of argyrodite after wet processing (Figure 3g).^[^ ^18^ ^]^

Tensile testing was conducted to evaluate SES mechanical suitability for roll‐to‐roll processing. While the h‐PI scaffold exhibits high stretchability due to its porous geometric structure, the coated SE layer exhibits significantly lower stretchability. As shown in Figure S7 (Supporting Information), stretching the SES beyond 6% strain causes the SE layer to crack and delaminate from the h‐PI scaffold due to differences in deformation behavior. Therefore, ultimate tensile strength alone is not a sufficient metric for scaffold‐supported SES evaluation.^[^ ^19^, ^20^, ^21^ ^]^ Instead, the practical tensile strength should be defined as the maximum stress at which the SE layer remains intact on the scaffold. As shown in Figure 3h, the SES developed in this work can withstand a tensile stress of 7.15 MPa at 6% strain without delamination, attributed to the excellent mechanical strength of the PI material. In contrast, the freestanding SE (FS‐SE) film, fabricated without a scaffold, exhibited an ultimate tensile strength of only 0.54 MPa at 5.8% strain and fractured at 6.3% strain. This substantial difference highlights the critical role of scaffold support in ensuring SES mechanical robustness for practical commercialization.

To further assess mechanical durability under flexural deformation, bending tests were conducted by repeatedly bending SES samples around glass vials of varying radii. As shown in Figure S8 (Supporting Information), the SES retained its ionic areal conductance with negligible degradation after 100 bending cycles, even at a minimum bending radius of 7.5 mm. This result confirms that both the scaffold and SE matrix retain their structural and functional integrity under repeated flexural stress, further supporting the SES's practical applicability in roll‐to‐roll manufacturing environments.

**Figure 4:**
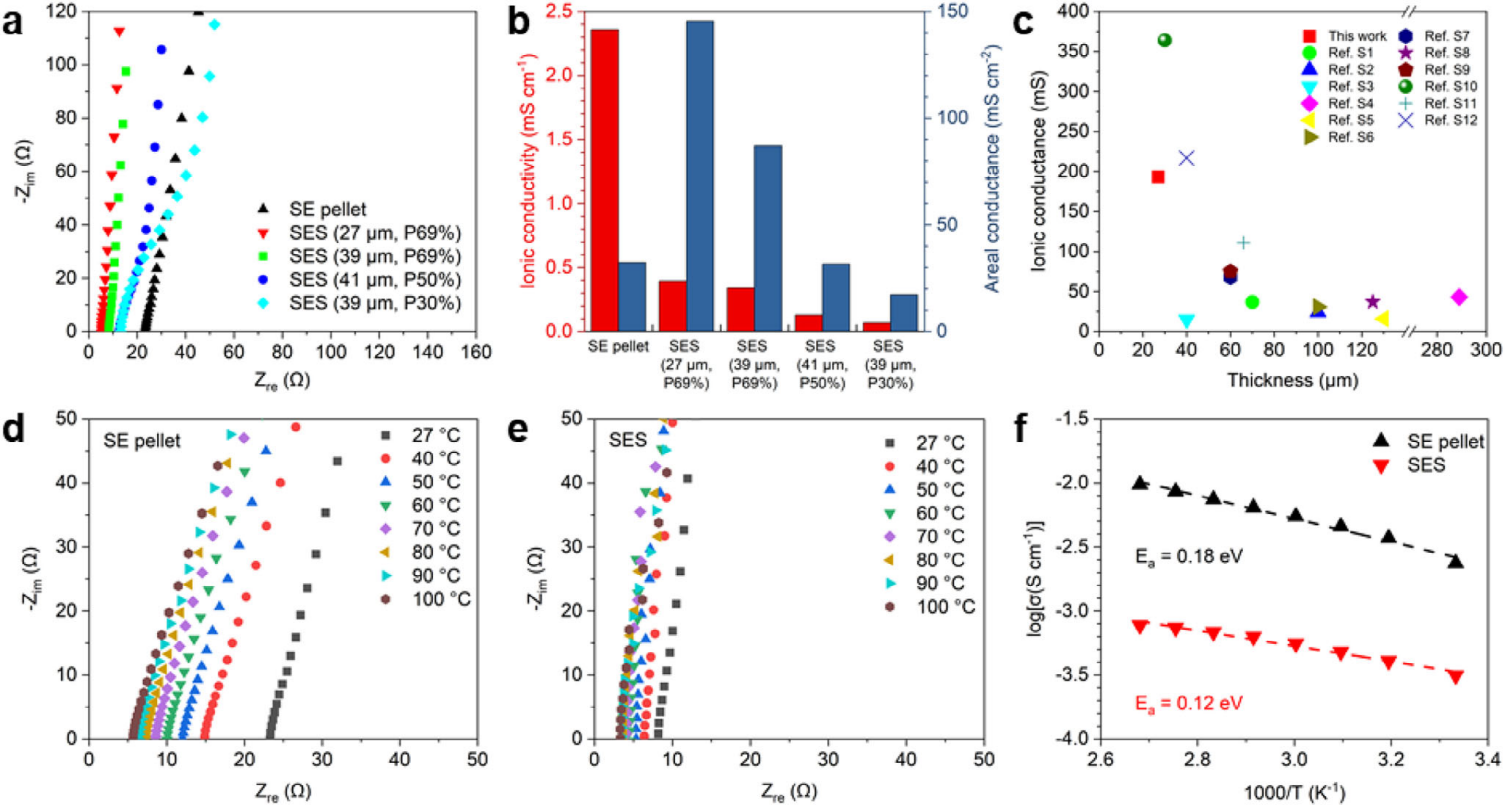
a) Nyquist plots of SE pellet and SES using h‐PI measured at 27 °C. b) Ionic conductivities and areal conductances are derived from the EIS data. c) Comparison of SES thickness and ionic conductance with other reported scaffold‐supported SE membranes. Nyquist plots of d) SE pellet and e) SES measured from 27 to 100 °C and f) corresponding Arrhenius plot of ionic conductivity.

The ionic transport properties of SESs were evaluated using electrochemical impedance spectroscopy (EIS) and compared with those of a conventional SE pellet. The Nyquist plots in **Figure** **4**a show a significantly lower impedance for SES (5.2 Ω) compared to the SE pellet (23.3 Ω), owing to the reduced thickness of the SES. To investigate the correlation between scaffold porosity and SES ionic conductivity, SESs with similar thicknesses were fabricated using h‐PI scaffolds with varying porosities, controlled by fixing the hole diameter at 200 µm and adjusting the gap distance. As shown in Figure 4b, SES ionic conductivity decreases with decreasing scaffold porosity due to increased ionic tortuosity, where Li ions must navigate a longer path around blocked scaffold regions. SESs fabricated using scaffolds with punched areas of 69%, 50%, and 30% exhibited ionic conductances of 87, 32, and 17 mS cm^−2^, respectively. The high porosity (69%) and thin scaffold (8 µm) enabled the 27 µm‐thick SES to achieve a high ionic conductance of 146 mS cm^−^^2^ (193 mS), significantly surpassing that of the SE pellet (32 mS cm^−^^2^). The SES developed in this study is the thinnest reported scaffold‐supported SES and exhibits one of the highest reported ionic conductance values to date (Figure 4c; Table S3, Supporting Information). In terms of ionic conductivity, the SE pellet and SES exhibited values of 2.36 and 0.393 mS cm^−1^, respectively, due to normalization with respect to thickness, which favors the denser structure of the SE pellet. The Nyquist plots and corresponding Arrhenius profiles of the SE pellet and SES at various temperatures are shown in Figure 4d–f.

**Figure 5:**
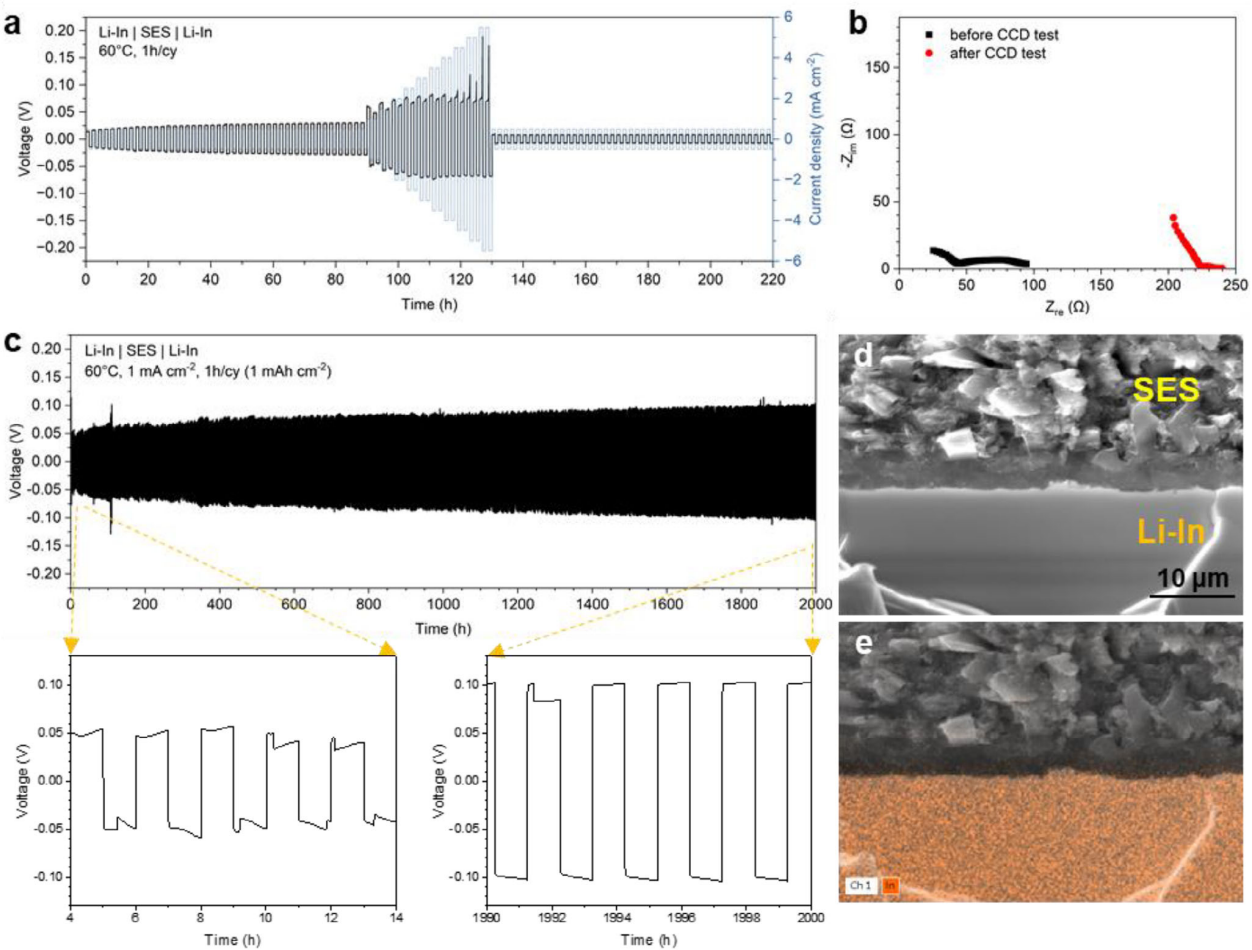
Electrochemical performance of Li‐In|SES|Li‐In symmetric cell at 60 °C. a) CCD test at various current densities (0.5–5.5 mA cm^−2^). b) Nyquist plots before and after the CCD test. c) Cyclic stability at 1 mA cm^−2^ per cycle for 1000 cycles. The enlarged voltage profiles are shown below. d) Cross‐sectional SEM image of Li‐In/SES interface after cycling. e) EDS mapping of In element distribution.

The critical current density (CCD) and cycling stability of SES were investigated using a Li‐In|SES|Li‐In symmetric cell at 60 °C. Li metal can creep under pressures as low as 0.8–2.4 MPa and penetrate even a ≈1 mm‐thick LPSCl pellet at a stack pressure of 75 MPa, leading to short‐circuiting.^[^ ^22^, ^23^ ^]^ This issue is more pronounced in thin SESs, making it challenging to ensure conformal contact at the Li/SE interface without inducing short circuits. To mitigate this, Li‐In was used as the anode in this study, despite its practical limitations, which is an issue that should be addressed in future research. As shown in **Figure** **5**a, the CCD test was conducted at various current densities ranging from 0.5 to 5.5 mA cm^−2^ for 1 h per cycle. The symmetric cell was initially cycled at 0.5 mA cm^−2^ for 45 cycles to stabilize the Li‐In/SES interface before gradually increasing the current density in 0.5 mA cm^−2^ increments. Notably, even after cycling at 5.5 mA cm^−2^ (equivalent to 1.83C with a 3 mAh cm^−2^ capacity cell), no short‐circuiting was observed, and the SES continued stable cycling at 0.5 mA cm^−2^. This exceptional stability at high current densities highlights the practical viability of the SES for high‐loading battery cells. EIS measurement after the CCD test further confirmed the absence of short‐circuit (Figure 5b). The high CCD in the Li‐In|SES|Li‐In symmetric cell can be attributed to the chemical/electrochemical stability of Li‐In against sulfide‐based SEs and the high lithium diffusivity in Li‐In, as previously reported in the literature.^[^ ^24^ ^]^ Furthermore, as shown in Figure 5c, the symmetric cell demonstrated stable cycling when subjected to galvanostatic cycling at 1 mA cm^−2^ for 1000 cycles, with a low overpotential of 50.2 mV, which increased to 102 mV after 2000 h of cycling. Cross‐sectional SEM and EDS images of the Li‐In/SES interface after 1000 cycles (Figure 5d,e) revealed a stable interphase with negligible contact loss. No detectable indium crossover into the SES was observed, further demonstrating the robustness of the SES interface.

**Figure 6:**
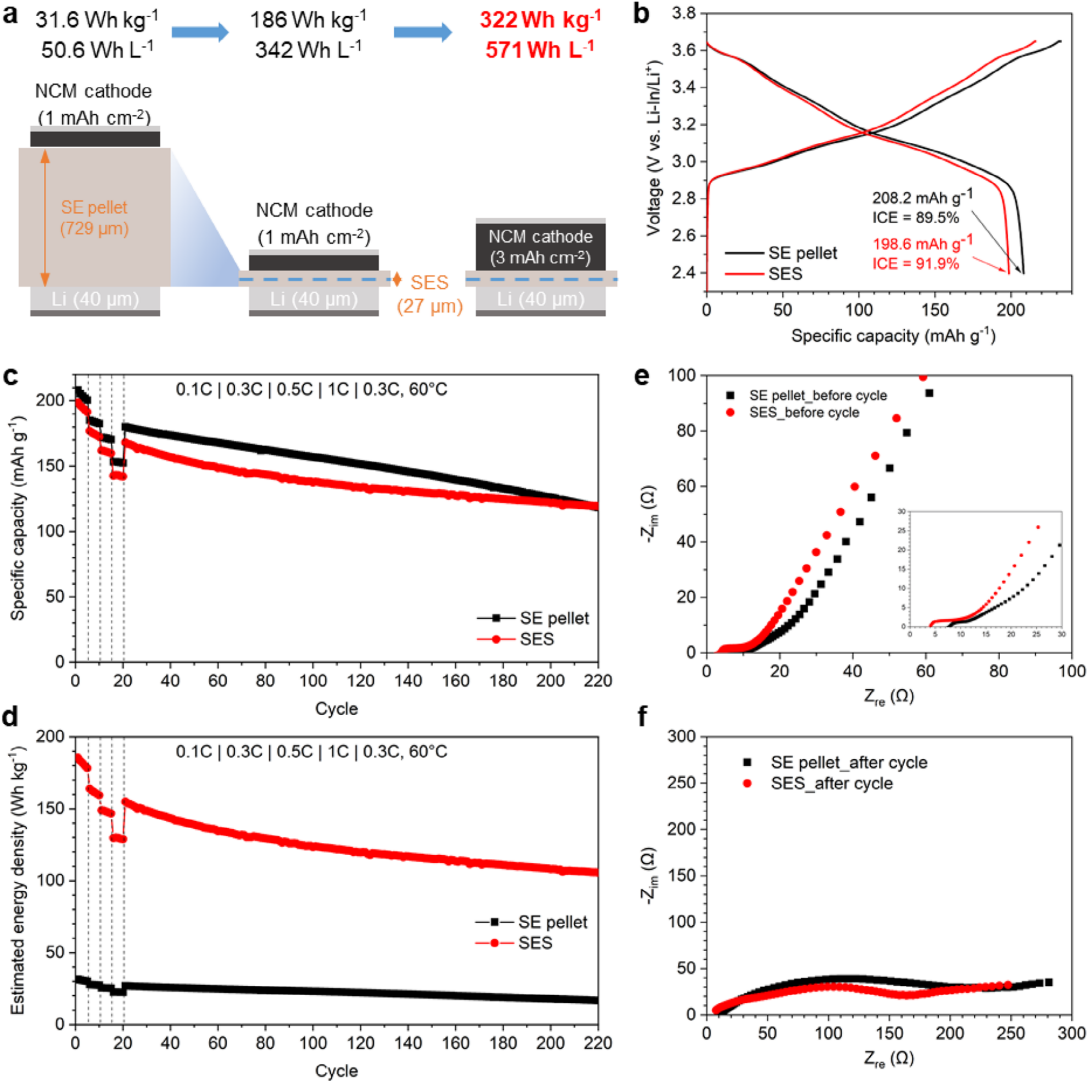
a) Schematic illustration of ASB cells with SE pellet or SES. b) Initial charge–discharge voltage profiles, c) rate capability, and cycle performance. d) Cycle performance in terms of cell‐level energy density. Nyquist plots e) before and f) after cycling. The inset in (e) shows the magnified Nyquist plot.

The electrochemical performance of LiNi_0.83_Co_0.11_Mn_0.06_O_2_ (NCM)||Li‐In half cells using the SE pellet and SES was evaluated and compared (**Figure** **6**a). A composite cathode with 80% active material composition and active material loading of 5.7 mg cm^−2^ (corresponding to an areal capacity of 1.1 mAh cm^−2^) was used to evaluate performance. As shown in the initial charge–discharge profiles (Figure 6b), the SE pellet and SES cells displayed similar discharge capacities of 208.2 and 198.6 mAh g^−1^, respectively, with initial Coulombic efficiencies (ICE) of 89.5% and 91.9%. The rate capability and cycle stability comparisons (Figure 6c) further indicate that both cells exhibited similar capacity retentions at 0.3C (88.9% vs 89.1%), 0.5C (82.6% vs 81.5%), and 1C (73.7% vs 71.8%), with long‐term cycling at 0.3C maintaining capacity retentions of 65.8% and 70.9% after 200 cycles for the SE pellet and SES cells, respectively. More importantly, the SES cell demonstrated significantly enhanced cell‐level energy densities (EDs) in both gravimetric and volumetric terms compared to the SE pellet cell due to its thinner electrolyte layer (Figure 6d). Assuming a 40 µm Li anode, the SE pellet cell exhibited gravimetric and volumetric EDs of 31.6 Wh kg_cell_^−1^ and 50.6 Wh L_cell_^−1^, respectively. In contrast, the SES cell exhibited substantially higher EDs of 186 Wh kg_cell_^−1^ and 342 Wh L_cell_^−1^, marking a significant advancement in ASB performance (Detailed information on cell components and ED calculations provided in the Tables S4 and S5, Supporting Information).

To gain deeper insight into the internal resistance of the cells, EIS measurements were conducted before and after cycling (Figure 6e,f), and the EIS spectra were fitted using an equivalent circuit model (Figure S9, Supporting Information). The resulting resistance contributions are summarized in Table S6 (Supporting Information). Three key differences emerged from the fitting results: First, the bulk resistance (R_b_) of the SES cell was lower than that of the SE pellet cell, reaffirming the higher ionic conductance of the SES, due to its thin structure. Second, the bulk resistance (R_b_) of the SES cell remained nearly unchanged after cycling (3.81 Ω → 3.78 Ω), whereas the SE pellet cell showed a modest increase (7.82 Ω → 8.55 Ω), suggesting more stable bulk ion transport in the SES structure. Third, the anode interfacial resistance (R_a_) in the high‐frequency range exhibited a more dramatic increase in the SE pellet cell (9.60 Ω → 97.2 Ω), compared to a smaller increase in the SES cell (4.17 Ω → 24.4 Ω). This difference is likely due to the limited mechanical compliance of the SE pellet cell. In the SE pellet cell, the rigid pellet becomes fixed to the mold wall during high‐pressure assembly, limiting its ability to accommodate volume changes during cycling. In contrast, the SES is pre‐pressed by CIP before assembly, and can accommodate the Li‐In anode volume changes, mitigating contact loss at the SE/Li‐In interface and minimizing structural damage to the SE layer. Meanwhile, the cathode interfacial resistance (R_c_) in the mid‐frequency range increased similarly in both cells, suggesting a comparable degree of interfacial evolution.

Furthermore, the SES cell demonstrated stable performance at room temperature of 27 °C (Figure S10, Supporting Information). Due to the lower ionic conductivity at 27 °C (≈1.8 times lower than at 60 °C), the SES cell delivered a lower initial discharge capacity of 176.4 mAh g^−1^ and an ICE of 82.8%. The capacity retentions at higher C‐rates of 0.3C, 0.5C, and 1C were 83.7%, 73.9%, and 59.9%, respectively. Nonetheless, despite the reduced ionic transport at room temperature, the SES cell maintained a higher cycle retention of 86.8% after 200 cycles at 0.3C, attributed to lower active material utilization and reduced contact loss during cycling.

**Figure 7:**
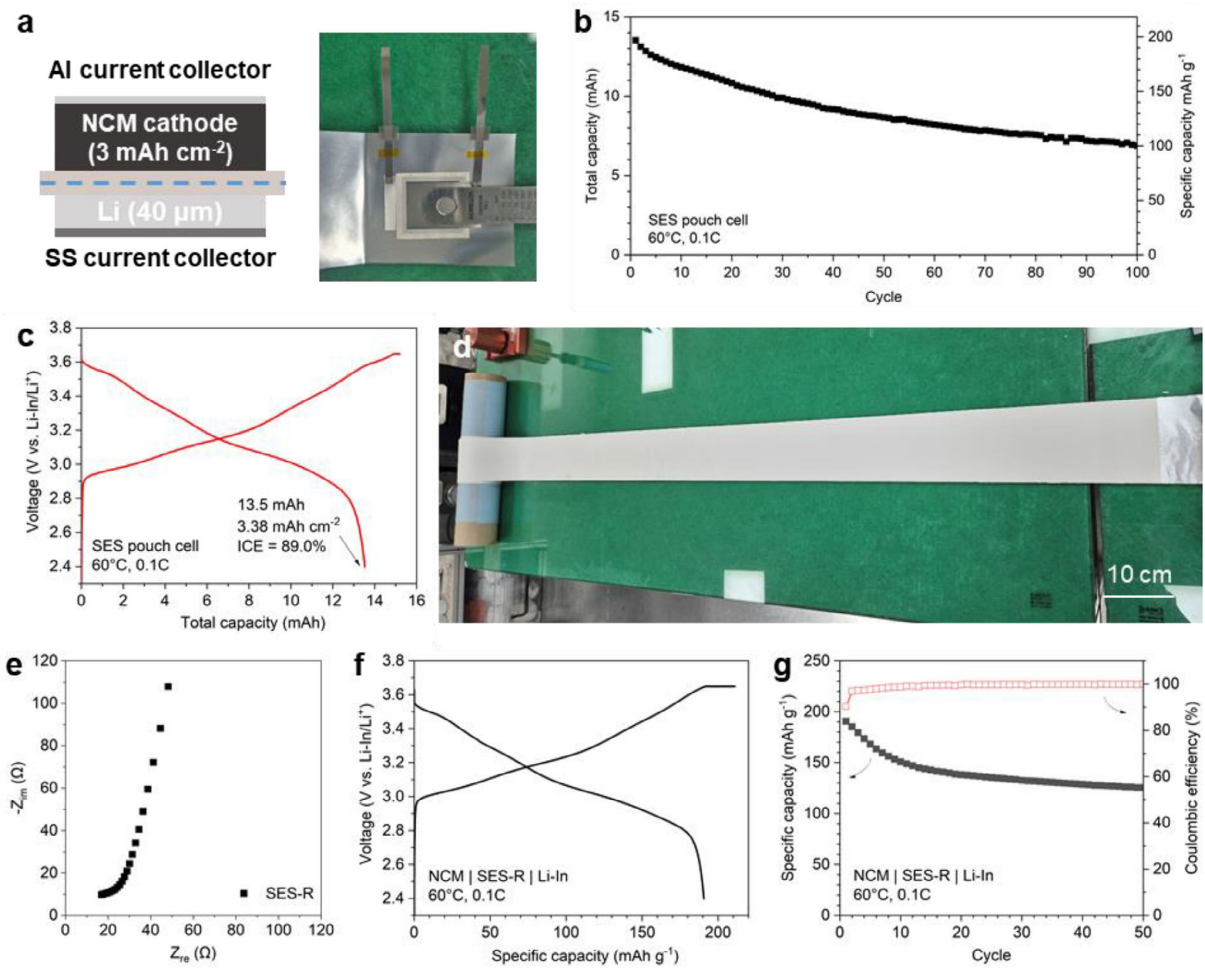
a) Schematic illustration and photograph of the assembled ASB pouch cell. b) Cycle performance and c) initial charge–discharge voltage profile. d) Photograph of the 4 m long SES roll (SES‐R) fabricated using a comma coater. e) EIS of SES‐R. f) Initial charge–discharge voltage profile and g) cycle performance of an ASB cell incorporating SES‐R.

To demonstrate practical viability, an ASB pouch cell was assembled with a higher cathode loading of 17.2 mg cm^−2^ (corresponding to an areal capacity of 3.4 mAh cm^−2^), as shown in **Figure** **7**a. The 13.5 mAh pouch exhibited an ICE of 89.0% and capacity retention of 50.9% after 100 cycles at 0.1C (Figure 7b,c; Figure S11a, Supporting Information). More importantly, the ASB pouch cell achieved high cell‐level EDs of 322 Wh kg_cell_^−1^ and 571 Wh L_cell_^−1^, which are among the highest reported values for ASBs to date and rival those of state‐of‐the‐art LIBs (detailed calculation shown in Tables S7 and S8, Supporting Information).^[^ ^5^, ^20^, ^25^, ^26^, ^27^, ^28^, ^29^, ^30^, ^31^, ^32^ ^]^ This substantial improvement arises from SES's thin structure and high ionic conductance, making it the thinnest reported SES with one of the highest ionic conductance values for scaffold‐supported SESs (Table S3, Supporting Information). The rate capability and room temperature cycle performance of the SES pouch cell were also evaluated, as shown in Figure S11b–d (Supporting Information). The SES pouch cell demonstrated stable room temperature cycle performance and maintained a cycle retention of 67.4% after 100 cycles at 0.1C.

To further demonstrate scalability, a 100 mm wide, 4 m long SES roll (SES‐R) was fabricated via roll‐to‐roll manufacturing using an industrial comma coater (Figure 7d; Figure S12, Supporting Information). The prototype SES‐R exhibited a thickness of 105 µm and areal conductance of 30 mS cm^−2^ (Figure 7e), and an ASB cell incorporating SES‐R displayed an initial discharge capacity of 190.7 mAh g^−1^, an ICE of 90.3%, and capacity retention of 65.8% after 50 cycles at 0.1C (Figure 7f,g).

**Figure 8:**
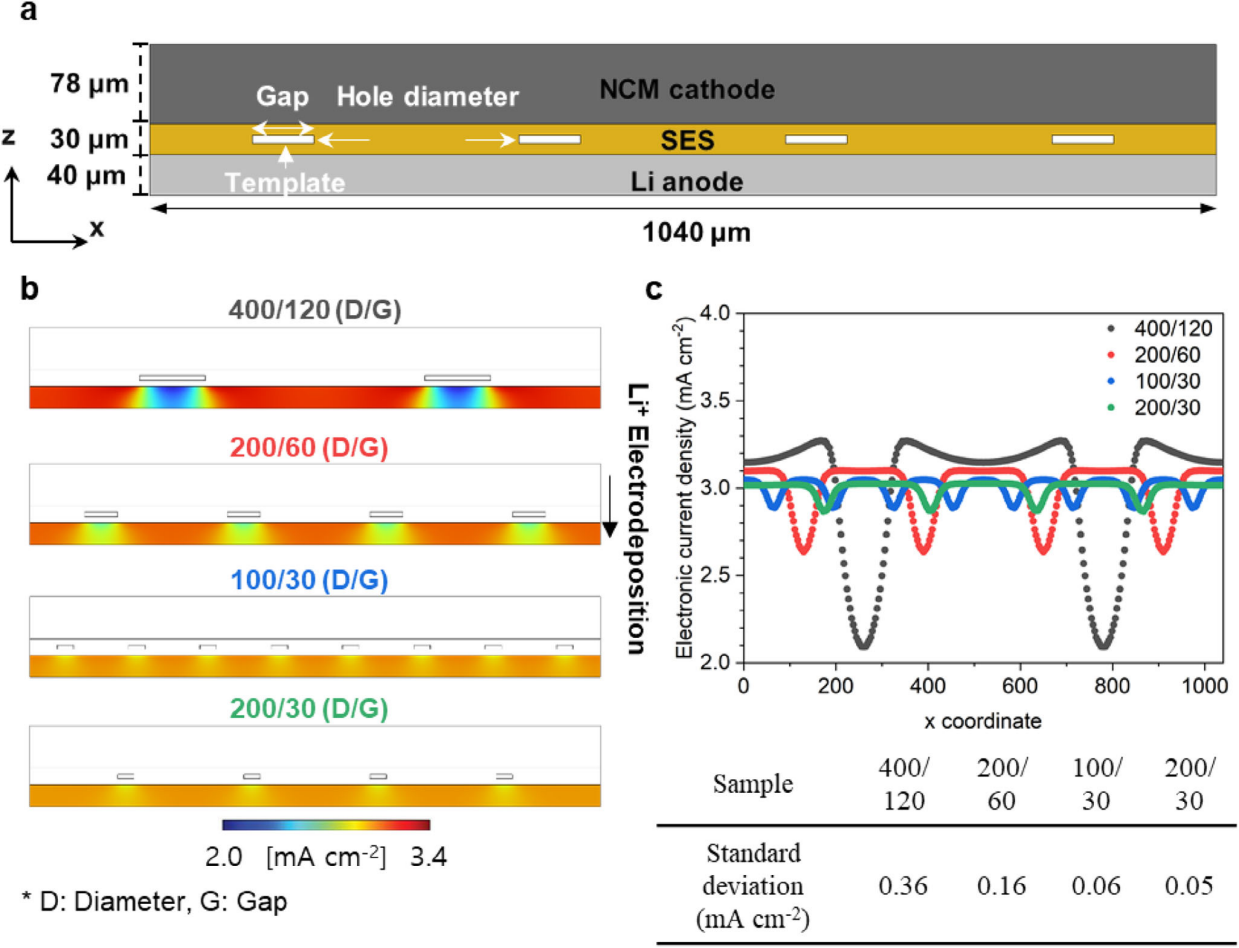
a) 2D simulation model geometry of an NCM|SES|Li full cell. b) Electronic current density distribution profile across the Li anode. c) Electronic current density profile at the SES/Li interface at the last moment (≒4.3 V) of constant current charging at 1C at 25 °C.

A potential concern with scaffold‐supported SES is non‐uniform Li‐ion flux distribution during electrochemical reactions due to the obstruction of Li‐ion transport by the scaffold material. To assess this, 2D models of the NCM|SES|Li full cell were constructed, as shown in **Figure** **8**a, and the electrochemical behavior under constant current charging was simulated. Details regarding the simulation conditions are provided in the Tables S9 and S10 (Supporting Information). To maximize the potential detrimental effect of the scaffold blockage, the simulation was conducted at room temperature (25°C) and a high C‐rate of 1C. As depicted in Figure 8b, the scaffold material impedes Li‐ion transport, leading to a non‐uniform current density distribution at the SES/Li interface. The electronic current density profile at the SES/Li interface (Figure 8c) indicates that the current density can vary significantly, ranging from 2.1 to 3.3 mA cm^−2^ for the 400/120 SES sample, corresponding to a standard deviation of 0.36 mA cm^−2^. However, reducing the hole diameter and gap distance improved uniformity. For example, the 100/30 SES sample, despite having the same 54% punched area, exhibited much‐improved uniformity (0.06 mA cm^−2^ standard deviation). The optimized SES structure (200/30) used in ASBs showed the most uniform Li‐ion flux (0.05 mA cm^−2^ standard deviation), confirming minimal hindrance from the scaffold. These simulation results provide a crucial guideline for future studies on scaffold‐supported SES to achieve uniform Li‐ion flux during electrochemical measurements.

To assess the broader applicability of our approach, SES incorporating Li_3_PS_4_ (LPS) as the SE (LPS‐SES) was also fabricated and tested. As shown in Figure S13a,b (Supporting Information), the LPS‐SES also demonstrated enhanced ionic conductance (25 mS cm^−2^) compared to its pellet counterpart (11 mS cm^−2^), attributed to its reduced thickness (34 µm). Symmetric cell test with Li‐In|LPS‐SES|Li‐In configuration (Figure S13c, Supporting Information) confirmed reversible Li plating/stripping behavior, indicating the feasibility of our scaffold‐supported approach for other sulfide‐based SEs. In addition, to evaluate the compatibility of the SES with alternative electrode chemistries, a natural graphite (NG)|SES|Li‐In half cell was also fabricated. As shown in Figure S13d,e (Supporting Information), the NG half cell exhibited stable and reversible capacities at various C‐rates, further demonstrating that the SES can be effectively integrated with carbonaceous anodes such as graphite.

## 3 Conclusion

This study demonstrates the development of a scaffold‐supported solid electrolyte separator (SES) using a thin, porous PI scaffold, highlighting its potential for high‐performance ASBs. The 27 µm thick SES, fabricated via a scalable tape casting method, exhibits exceptional mechanical robustness (tensile stress of 7.15 MPa at 6% strain), high flexibility, and superior ionic conductance of 146 mS cm^−2^, effectively addressing the limitations of conventional SE pellets. The thin SES enables substantial enhancements in cell‐level energy density (ED), achieving estimated gravimetric and volumetric EDs of 322 Wh kg^−^¹ and 571 Wh L^−^¹, respectively. These values greatly surpass those of SE pellet‐based cells and are comparable to those of state‐of‐the‐art lithium‐ion batteries (LIBs). Furthermore, the optimized scaffold design (Tri_D200_G30_P69, featuring 69% porosity and uniform pore distribution) was identified as a key factor in ensuring uniform Li‐ion flux, thereby minimizing ionic transport heterogeneity and mitigating the risk of non‐uniform Li plating. Simulation studies further confirmed the significance of pore structure optimization, revealing that a well‐distributed porous network enhances electrochemical performance and cycle stability by preventing localized current density fluctuations. Additionally, the practical scalability of the proposed scaffold‐supported SES was demonstrated through the fabrication of a 100 mm wide, 4 m long SES roll (SES‐R) via roll‐to‐roll manufacturing, confirming its commercial feasibility.

## 4 Experimental Section

### Laser Drilling of Scaffolds

Laser drilling was conducted with a UV laser drilling system (ESI, 5330he). Polyimide (PI) films with thicknesses of 8 and 12 µm and Ni foil with a thickness of 10 µm were used as the template.

### Fabrication of SES, NCM Composite Cathode, Li‐In Anode, and NG Composite Anode

Solid electrolyte (SE) slurry was prepared by first dissolving nitrile butadiene rubber (NBR; LG Energy Solution) in anhydrous anisole (Sigma–Aldrich, 99.7%). Argyrodite Li_6_PS_5_Cl (LPSCl) powder (POSCO JK Solid Solution) with D_50_ particle size of 3 µm was then dispersed into the NBR solution with a LPSCl:NBR mass ratio of 99:1 using a planetary mixer (Thinky Corp., ARE‐310). The resulting SE slurry was coated onto the laser‐drilled scaffold, which was priory attached flat to a polytetrafluoroethylene (PTFE) delamination film, using the tape casting method and dried under vacuum at 60 °C overnight. For double‐side coating, the same tape casting procedure was applied to the backside after coating the SE slurry on one side. The same slurry composition and fabrication protocol were used to manufacture a large‐area SES (SES‐L) using a comma coater, except that a porous Al scaffold (Woori Science) was used for SES‐L fabrication. The same fabrication protocol was used to manufacture LPS‐SES, except that Li_3_PS_4_ (LPS) powder (POSCO JK Solid Solution) was used instead of LPSCl.

Cathode slurry was prepared by mixing LiNi_0.83_Co_0.11_Mn_0.06_O_2_ (NCM; LG Energy Solution), LPSCl, and Super P Li powders in NBR solution using a planetary mixer. The composition of the cathode slurry consisted of a mass ratio of 80:18:2:1 for NCM:LPSCl: Super P Li:NBR. NCM was coated with 0.5 wt.% LiNbO_3_ (LNO) prior to making the cathode slurry using the procedures reported previously in the literature.^[^ ^33^ ^]^ The cathode slurry was coated onto an aluminum foil using the tape casting method and dried under vacuum at 100 °C overnight. The mass loadings of NCM active material were ≈6 and ≈18 mg cm^−2^, corresponding to areal capacities of 1.2 and 3.6 mAh cm^−2^, respectively.

Li‐In alloy anode was prepared by stacking Li foil (40 µm, Honjo Metal Co.) and In foil (100 µm, Sigma–Aldrich) and pressing them together using cold isostatic pressing (CIP) at 500 MPa.

Natural graphite (NG) composite anode was prepared by mixing NG (LG Chem) and LPSCl powders in NBR solution using a planetary mixer. The resulting composite slurry, with a mass ratio of NG:LPSCl:NBR = 60:38:2, was coated onto a stainless steel (SS) foil using the tape casting method and dried under vacuum at 100 °C overnight. The mass loading of NG active material was 7.8 mg cm^−2^, corresponding to an areal capacity of 2.8 mAh cm^−2^.

All procedures were conducted in a dry room with a moisture level maintained under 20 ppm.

### ASB Cell Assembly

Li‐In|SES|Li‐In symmetric cell was assembled by cold pressing the components at 300 MPa, using an in‐house pressure mold. Constant stack pressure of 20 MPa was applied to the assembled cell throughout the Li stripping/plating test.

NCM|SES|Li‐In half cell was assembled by first pressing NCM composite cathode and SES using CIP at 500 MPa. The pressed composite was then punched into desired dimensions, and a Li‐In anode was attached to the opposite side of the SES. The cell was subsequently cold‐pressed at 300 MPa using an in‐house pressure mold. A constant stack pressure of 20 MPa was applied to the assembled cell throughout the electrochemical measurements. The NG half cell and ASB pouch cell were assembled following the same protocol.

### Material Characterization

Material morphologies were examined with a scanning electron microscope (SEM) (SEC, SNE‐4500M) equipped with an energy‐dispersive X‐ray spectroscopy detector (EDS) (Bruker, XFlash 640H Mini) and an optical microscope (OM) (Olympus, BX53MRF). X‐ray diffraction (XRD) (Malvern Panalytical, Aeris) measurements were conducted by sealing the samples with polyimide film in an argon glovebox before measurement. Raman spectra were acquired with high resolution Raman/PL system (Horiba, LabRAM HR Evolution) using a 514 nm source.

### Tensile and Bending Tests

Tensile strength was measured using a tensile test machine with a testing speed of 0.10 mm s^−1^. Bending durability was evaluated by repeatedly bending 2 × 2 cm^2^ SES samples around glass vials with radii of 21.0, 13.7, and 7.5 mm for 100 cycles. The ionic areal conductance of the SES was measured using electrochemical impedance spectroscopy (EIS) following the bending test.

### Electrochemical Measurements

The ionic conductivities/conductances of the SES and SE pellet were evaluated using EIS (Biologic, SP‐150) at an amplitude of 10 mV and frequency range of 100 kHz–50 mHz. SESs were cold‐pressed at 440 MPa using a titanium pressure mold prior to testing. Galvanostatic cycling tests were conducted using a cycle tester in a 60 °C chamber (Toyo System, TOSCAT). For symmetric Li‐In||Li‐In cell test, Li stripping/plating was conducted at a current density of 1 mA cm^−2^ for 1 h. For half cell test, constant current/constant voltage (CC/CV) and constant current (CC) modes were used for discharge and charge, respectively. Cut‐off voltage was set at 2.4 and 3.7 V, and the cut‐off current in the CV mode was set at one‐fifth of the original current.

### Electrochemical Simulation Workflow

Charging simulations were conducted using the Li‐ion Battery module in COMSOL Multiphysics 6.2, a finite element method‐based commercial simulation package, with four 2D geometries (D400_G120, D200_G60, D100_G30, and D200_G30). The detailed workflow of the current density simulation was described in our previous works.^[^ ^34^, ^35^ ^]^ Design and electrochemical parameters utilized in full cell simulations are presented in the Supporting Information, respectively. All calculations were done with an AMD Ryzen Threadripper PRO 5995WX running at 4.00 GHz with 1012 GB of memory.

## Conflict of Interest

The authors declare no conflict of interest.

## Supporting information



Supporting Information

## Data Availability

The data that support the findings of this study are available from the corresponding author upon reasonable request.

## References

[1] T. Liu , Y. Yuan , X. Tao , Z. Lin , J. Lu , Adv. Sci. 2020, 7, 2001207. 10.1002/advs.202001207 PMC7507509 32995126

[2] Q. Zhang , D. Cao , Y. Ma , A. Natan , P. Aurora , H. Zhu , Adv. Mater. 2019, 31, 1901131. 10.1002/adma.201901131 31441140

[3] K. J. Kim , M. Balaish , M. Wadaguchi , L. Kong , J. L. M. Rupp , Adv. Energy Mater. 2021, 11, 2002689.

[4] Y.‐G. Lee , S. Fujiki , C. Jung , N. Suzuki , N. Yashiro , R. Omoda , D.‐S. Ko , T. Shiratsuchi , T. Sugimoto , S. Ryu , J. H. Ku , T. Watanabe , Y. Park , Y. Aihara , D. Im , I. T. Han , Nat. Energy 2020, 5, 299.

[5] H. Liu , Y. Liang , C. Wang , D. Li , X. Yan , C. W. Nan , L. Z. Fan , Adv. Mater. 2023, 35, 2206013. 10.1002/adma.202206013 35984755

[6] J. M. Whiteley , P. Taynton , W. Zhang , S.‐H. Lee , Adv. Mater. 2015, 27, 6922. 26421754 10.1002/adma.201502636

[7] D. Y. Oh , Y. J. Nam , K. H. Park , S. H. Jung , K. T. Kim , A. R. Ha , Y. S. Jung , Adv. Energy Mater. 2019, 9, 1802927.

[8] C. Wang , R. Yu , H. Duan , Q. Lu , Q. Li , K. R. Adair , D. Bao , Y. Liu , R. Yang , J. Wang , S. Zhao , H. Huang , X. Sun , ACS Energy Lett. 2022, 7, 410.

[9] Y. B. Song , K. H. Baeck , H. Kwak , H. Lim , Y. S. Jung , Adv. Energy Mater. 2023, 13, 2301142.

[10] Y. J. Nam , S.‐J. Cho , D. Y. Oh , J.‐M. Lim , S. Y. Kim , J. H. Song , Y.‐G. Lee , S.‐Y. Lee , Y. S. Jung , Nano Lett. 2015, 15, 3317. 25919229 10.1021/acs.nanolett.5b00538

[11] D. H. Kim , Y.‐H. Lee , Y. B. Song , H. Kwak , S.‐Y. Lee , Y. S. Jung , ACS Energy Lett. 2020, 5, 718.

[12] S. Yubuchi , W. Nakamura , T. Bibienne , S. Rousselot , L. W. Taylor , M. Pasquali , M. Dollé , A. Sakuda , A. Hayashi , M. Tatsumisago , J. Power Sources 2019, 417, 125.

[13] D. H. Kim , D. Y. Oh , K. H. Park , Y. E. Choi , Y. J. Nam , H. A. Lee , S.‐M. Lee , Y. S. Jung , Nano Lett. 2017, 17, 3013. 28362097 10.1021/acs.nanolett.7b00330

[14] A. Tron , A. Paolella , A. Beutl , Batteries 2023, 9, 503.

[15] S. H. Kang , J. Choi , J. Y. Kim , D. O. Shin , Y.‐G. Lee , J. Lee , ACS Appl. Mater. Interfaces 2023, 15, 28064. 37218997 10.1021/acsami.3c03466

[16] D. R. Rajagopalan Kannan , P. K. Terala , P. L. Moss , M. H. Weatherspoon , Int. J. Electrochem. 2018, 2018, 1.

[17] L. Zhou , K.‐H. Park , X. Sun , F. Lalère , T. Adermann , P. Hartmann , L. F. Nazar , ACS Energy Lett. 2019, 4, 265.

[18] Y. Wang , H. Hao , K. G. Naik , B. S. Vishnugopi , C. D. Fincher , Q. Yan , V. Raj , H. Celio , G. Yang , H. Fang , Y. M. Chiang , F. A. Perras , P. Jena , J. Watt , P. P. Mukherjee , D. Mitlin , Adv. Energy Mater. 2024, 14, 2304530.

[19] G. L. Zhu , C. Z. Zhao , H. J. Peng , H. Yuan , J. K. Hu , H. X. Nan , Y. Lu , X. Y. Liu , J. Q. Huang , C. He , J. Zhang , Q. Zhang , Adv. Funct. Mater. 2021, 31, 2101985.

[20] H. Liu , P. He , G. Wang , Y. Liang , C. Wang , L.‐Z. Fan , Chem. Eng. J. 2022, 430, 132991.

[21] D. Li , H. Liu , C. Wang , C. Yan , Q. Zhang , C. W. Nan , L. Z. Fan , Adv. Funct. Mater. 2024, 34, 2315555.

[22] A. Masias , N. Felten , R. Garcia‐Mendez , J. Wolfenstine , J. Sakamoto , J. Mater. Sci. 2019, 54, 2585.

[23] J. M. Doux , H. Nguyen , D. H. S. Tan , A. Banerjee , X. Wang , E. A. Wu , C. Jo , H. Yang , Y. S. Meng , Adv. Energy Mater. 2020, 10, 1903253.

[24] W. J. Jeong , C. Wang , S. G. Yoon , Y. Liu , T. Chen , M. T. McDowell , ACS Energy Lett. 2024, 9, 2554. 38903403 10.1021/acsenergylett.4c00915 PMC11187630

[25] J. Betz , G. Bieker , P. Meister , T. Placke , M. Winter , R. Schmuch , Adv. Energy Mater. 2019, 9, 1803170.

[26] Y. Lu , X. Rong , Y.‐S. Hu , L. Chen , H. Li , Energy Storage Mater. 2019, 23, 144.

[27] R. Xu , J. Yue , S. Liu , J. Tu , F. Han , P. Liu , C. Wang , ACS Energy Lett. 2019, 4, 1073.

[28] W. Jiang , L. Yan , X. Zeng , X. Meng , R. Huang , X. Zhu , M. Ling , C. Liang , ACS Appl. Mater. Interfaces 2020, 12, 54876. 33236875 10.1021/acsami.0c17828

[29] T. Jiang , P. He , Y. Liang , L.‐Z. Fan , Chem. Eng. J. 2021, 421, 129965.

[30] Y. Wang , J. Ju , S. Dong , Y. Yan , F. Jiang , L. Cui , Q. Wang , X. Han , G. Cui , Adv. Funct. Mater. 2021, 31, 2101523.

[31] T. Yersak , J. R. Salvador , R. D. Schmidt , M. Cai , Int. J. Appl. Glass Sci. 2021, 12, 124.

[32] S. Liu , L. Zhou , J. Han , K. Wen , S. Guan , C. Xue , Z. Zhang , B. Xu , Y. Lin , Y. Shen , L. Li , C. W. Nan , Adv. Energy Mater. 2022, 12, 2200660.

[33] N. Ohta , K. Takada , I. Sakaguchi , L. Zhang , R. Ma , K. Fukuda , M. Osada , T. Sasaki , Electrochem. Commun. 2007, 9, 1486.

[34] J. Park , D. Kim , D. Jin , C. Phatak , K. Y. Cho , Y.‐G. Lee , S. Hong , M.‐H. Ryou , Y. M. Lee , J. Power Sources 2018, 408, 136.

[35] J. Park , J. Jeong , Y. Lee , M. Oh , M.‐H. Ryou , Y. M. Lee , Adv. Mater. Interfaces 2016, 3, 1600140.

