# Supplementary material for: High‐Performance, Roll‐to‐Roll Fabricated Scaffold‐Supported Solid Electrolyte Separator for Practical All‐Solid‐State Batteries
Source: Small. 2025 Jul 1;21(38):2502996. doi: 10.1002/smll.202502996 (PMC12462554; doi:10.1002/smll.202502996)
Supplement: Supplementary file 1 — Supporting Information [file SMLL-21-2502996-s001.docx]

High-Performance, Roll-to-Roll Fabricated Scaffold-Supported Solid Electrolyte Separator for Practical All-Solid-State Batteries

*Seok Hun Kang^a^, Hyobin Lee^b^, Young-Jin Hong^c^, Seokhan Myoung^c^, Hyewon Seo^c^, Jaecheol Choi^a^, Seokyoon Yoon^a^, Ju Young Kim^a^, Dong Ok Shin^a^, Myeong Ju Lee^a^, Young-Sam Park^a^, Young-Gi Lee^a,*^, Yong Min Lee^b,d,*­­^*

^a^ Materials and Components Research Division, Electronics and Telecommunications Research Institute (ETRI), 218 Gajeongno, Yuseong-gu, Daejeon 34129, Republic of Korea

^b^ Department of Energy Science and Engineering, Daegu Gyeongbuk Institute of Science and Technology (DGIST), 333 Techno Jungang-daero, Dalseong-gun, Daegu 42988, Republic of Korea

^c^ R&D Center, MinTech Co., Ltd, 62 Gukjegwahak 21-ro, Yuseong-gu, Daejeon 34002, Republic of Korea

^d^ Department of Chemical and Biomolecular Engineering, Yonsei University, 50 Yonsei-ro, Seodaemun-gu, Seoul 03722, Republic of Korea

* Corresponding author e-mail: yongmin@yonsei.ac.kr

* Corresponding author e-mail: [lyg@etri.re.kr](mailto:lyg@etri.re.kr)

*Energy density (ED) calculations*

The total energy (Wh) stored in the battery was calculated by integrating the first discharge curve of the battery cell. The average voltage was determined by dividing the total energy by the discharge capacity. To account for the difference in redox potential between Li-In (0.62 V vs. Li^+^/Li) and Li, 0.62 V was added to the average voltage. This adjusted voltage was then multiplied by the discharge capacity to obtain the hypothetical energy (Wh) of a cell, assuming Li as the anode. The gravimetric and volumetric EDs were calculated by dividing the hypothetical energy by the total mass and volume of the cathode, SE layer (SE pellet or SES), a 40 μm thick Li anode, Al current collector, and stainless steel (SS) current collector.

**
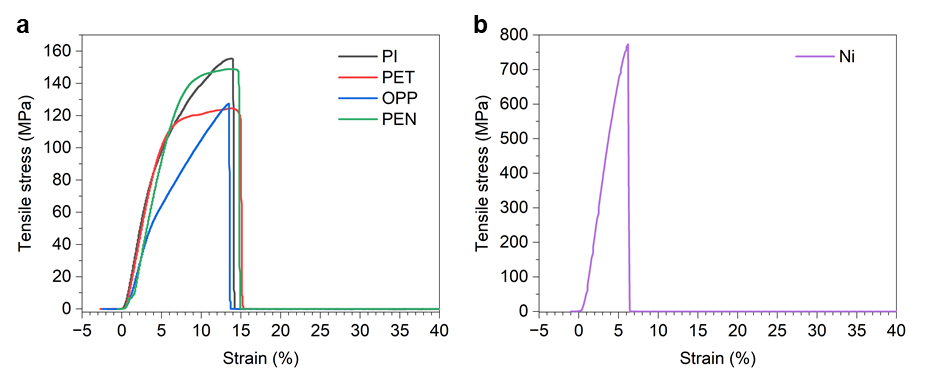
**

**Figure S1**. Tensile stress-strain curves of (a) various polymer films and (b) pristine Ni foil.

Table S1. Parameters of laser-drilled hole arrays and corresponding punched areas.

| Hole diameter (μm) | Gap (μm) | Punched area (%) | |
| --- | --- | --- | --- |
|  |  | Square array | Triangular array |
| 100 | 30 | 46 | 54 |
| 200 | 60 | 46 | 54 |
| 400 | 120 | 46 | 54 |
| 200 | 30 | 59 | 69 |
| 400 | 60 | 59 | 69 |
| 400 | 30 | 68 | 78 |


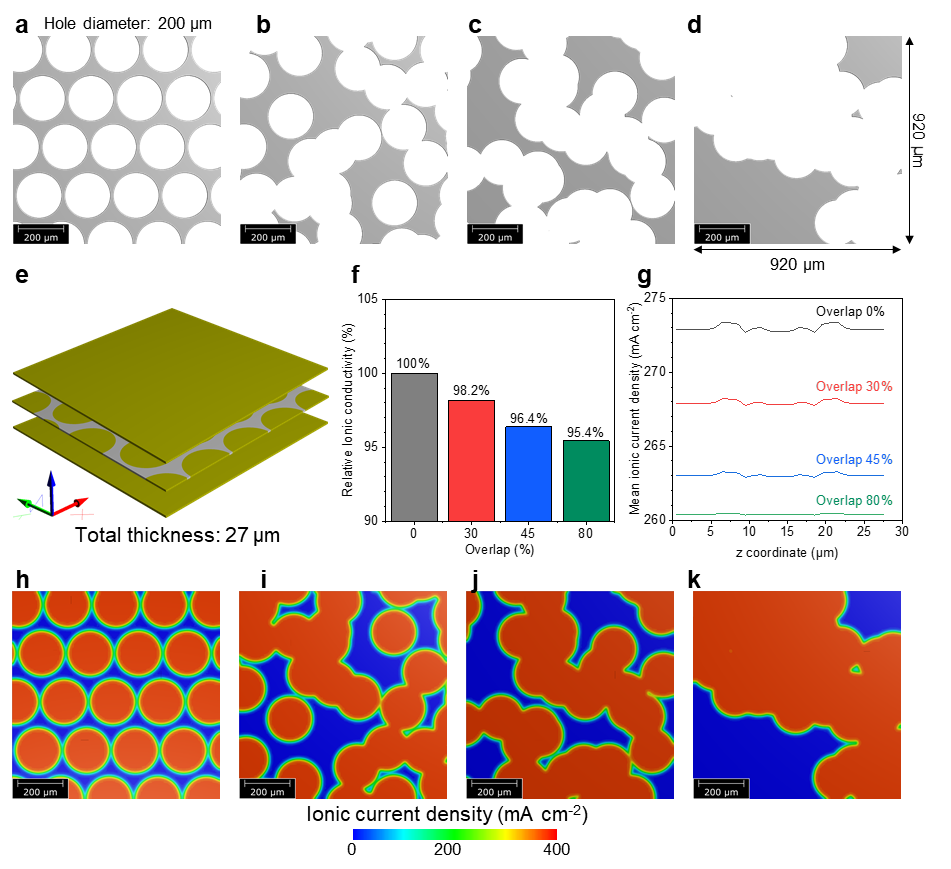


**Figure S2**. Scaffold structures with varying degrees of hole overlap: (a) 0%, (b) 30%, (c) 45%, and (d) 80% overlap. (e) Illustration of SES structure incorporating the porous scaffold. (f) Relative ionic conductivity values and (g) mean ionic current density in the thickness direction, calculated from ionic transport simulations. (h-k) Top-view images of simulated ion flux for each scaffold structure.


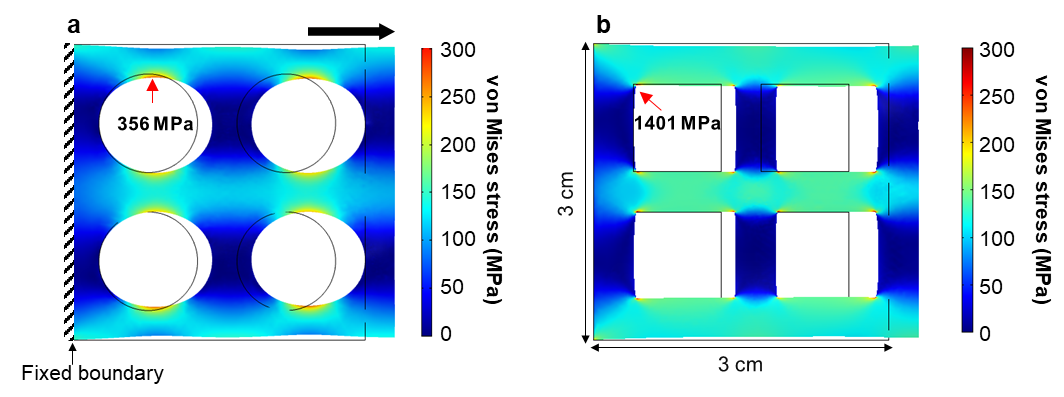


**Figure S3**. Tensile simulation results of scaffolds featuring (a) round- and (b) square-shaped holes under 10% tensile strain.

**
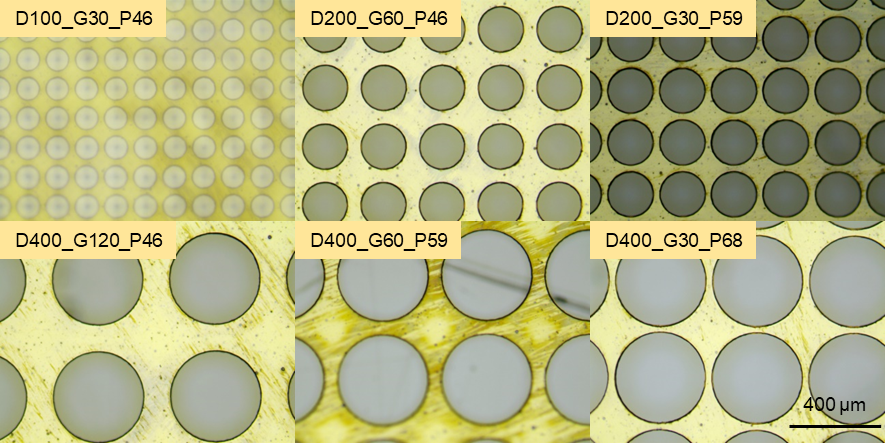
**

**Figure S4**. OM images of laser-drilled h-PI film, with hole diameter X μm, gap distance Y μm, and corresponding punched area Z%, in a square array.


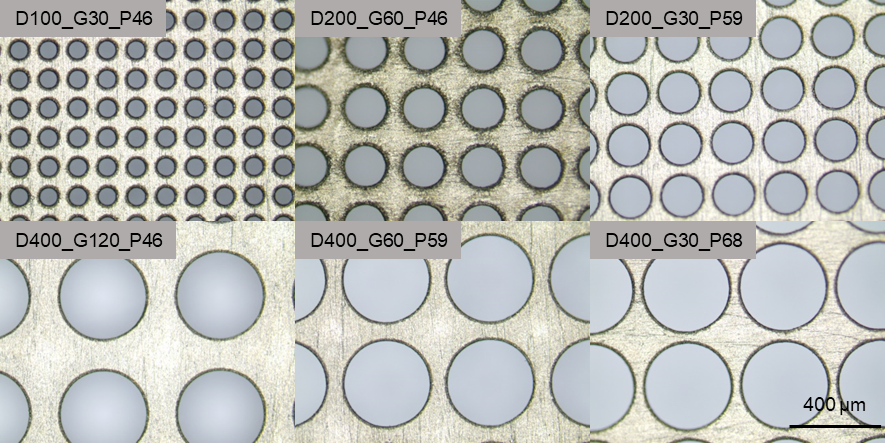


**Figure S5**. OM images of laser-drilled h-Ni foil, with hole diameter X μm, gap distance Y μm, and corresponding punched area Z%, in a square array.

Table S2. Conversion of tensile force to tensile strength for SESs required for roll-to-roll processing.

| Sample thickness (μm) | Cross-sectional area (mm^2^) | Tensile force (N) | Tensile strength (MPa) |
| --- | --- | --- | --- |
| 30 | 9.0 | 20 | 2.2 |
|  |  | 30 | 3.3 |
|  |  | 40 | 4.4 |
|  |  | 50 | 5.6 |
|  |  | 60 | 6.7 |
|  |  | 70 | 7.8 |
| 20 | 6.0 | 20 | 3.3 |
|  |  | 30 | 5.0 |
|  |  | 40 | 6.7 |
|  |  | 50 | 8.3 |
|  |  | 60 | 10.0 |
|  |  | 70 | 11.7 |
| 15 | 4.5 | 20 | 4.4 |
|  |  | 30 | 6.7 |
|  |  | 40 | 8.9 |
|  |  | 50 | 11.1 |
|  |  | 60 | 13.3 |
|  |  | 70 | 15.6 |

* Sample width assumed constant at 30 cm.


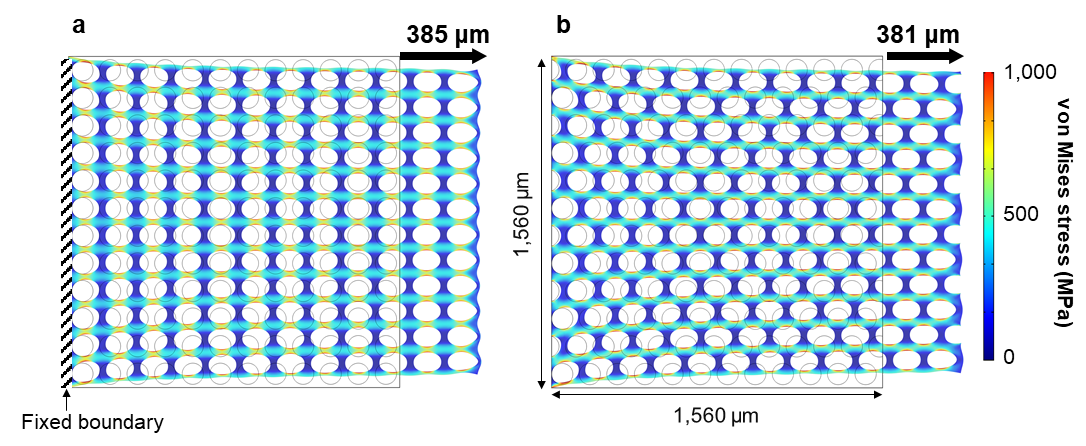


**Figure S6**. Tensile simulation results of scaffolds with (a) square and (b) triangular hole array patterns, subjected to a constant tensile force of 2 N.


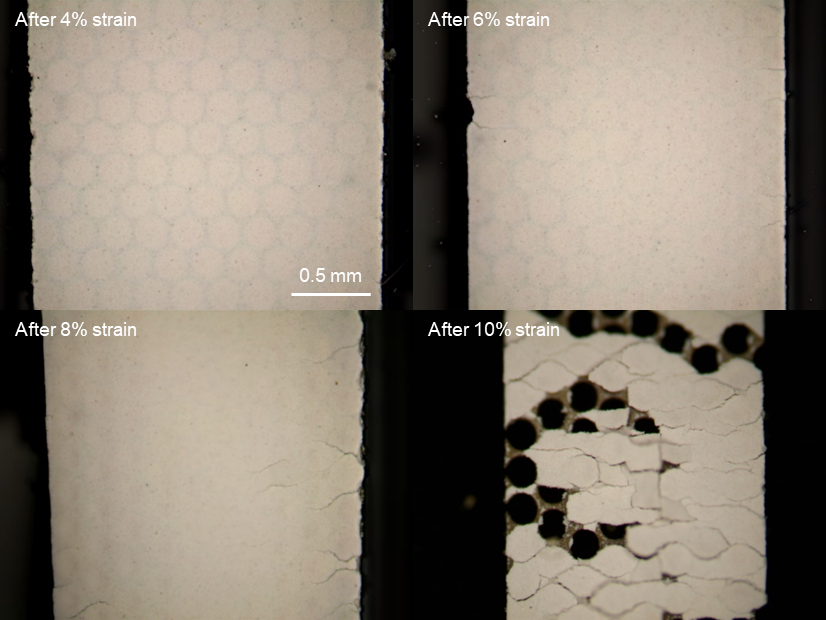


**Figure S7**. OM images of SES after applying various strain ranging from 4~10%.


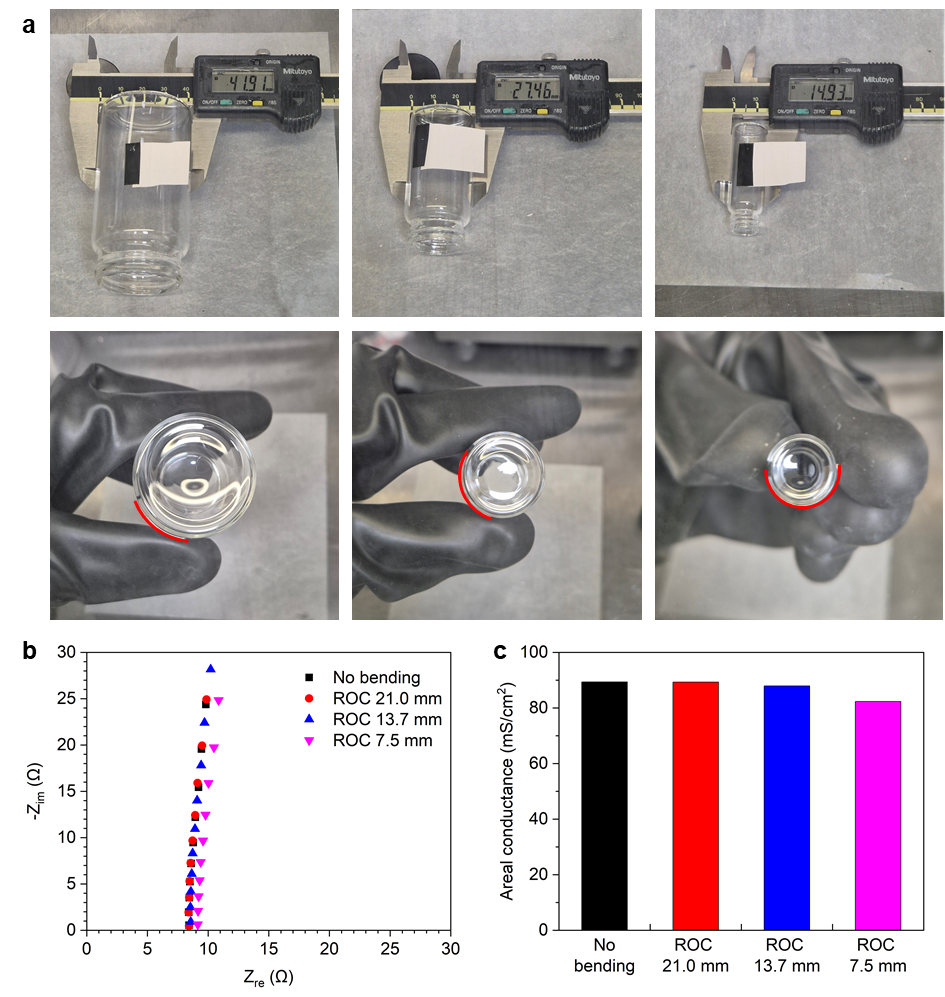


**Figure S8.** (a) Photograph of SES bending test at various radius of curvature (ROC). (b) Nyquist plots and (c) corresponding areal conductances of SES after bending test.

Table S3. Comparison of this work with other reported SES using porous scaffolds.

| SES | Thickness  (μm) | Ionic conductance  (mS cm^-2^)^**^ | Cell config. | Cell type | Active material loading level (mg cm^-2^) | Operating  temp (°C) | Discharge cap (mAh g^-1^) | Ref. |
| --- | --- | --- | --- | --- | --- | --- | --- | --- |
|  |  |  |  |  |  |  |  |  |
| Li_3_PS_4_ on PPTA | 70 | 28  (37 mS) | LCO/LTO | Pressure | 7.9 | 30 | 85 | ^[1]^ |
| Li_3_PS_4_ on Kevlar | 100 | 30 (24 mS) | Li_2_S/Li | Pressure | 2.54 | 25 | 1070.1 | ^[2]^ |
| Li_6_PS_5_Cl_0.5_Br_0.5_  on PI nonwoven | 40 | 15  (15 mS) | NCM622/Gr | Pressure | 8.5 | 30 | 146 | ^[3]^ |
| Li_10_GeP_2_S_12_  on EVAP | 289 | 55  (43 mS) | NCM333/Li | Coin | 2.94 | 50 | 102.6 | ^[4]^ |
| LiPSiS on non-woven fiberglass | 130~180 | (16 mS) | S/Li | Pouch | -  (2.6 mAh cm^-2^) | RT | - | ^[5]^ |
| Li_10_GeP_2_S_12_/PTFE  on Nylon | 100 | 39 (31 mS) | NCM532/GPE-Li | Pressure | 6 | RT | 138 | ^[6]^ |
| Li_6_PS_5_Cl on cellulose | 60 | (68 mS) | LTO/Li | Pouch | 3.2 | 25 | 148 | ^[7]^ |
|  |  |  | S/Li-In |  | 2.5 |  | 1064 |  |
| p-Li_6_PS_5_Cl/ P(PEGMEA) | 125 | (37 mS) | NCM811/Li | Pressure | 1.89 | RT | 157 | ^[8]^ |
| Li_10_GeP_2_S_12_/PEO/CTMS on Nylon | 60 | (75 mS) | NCM622/Gel-Li | Pressure | - | 25 | 142.4 | ^[9]^ |
| Li_6_PS_5_Cl on P(VDF-TrFE) | 30~40 | (364 mS) | NCM811/ Li-In | Coin | 3.1 | RT | 189 | ^[10]^ |
|  |  |  |  | Pouch | 7.4 |  | 189 |  |
| Li_6_PS_5_Cl/NBR on Nylon | 66 | 84  (111 mS) | LCO/Li | Pressure | 2.0 | 60 | 120 | ^[11]^ |
|  |  |  | NCM/Li | Pouch | 5.3 |  | 160 |  |
| Li_5.7_PS_4.7_Cl_1.3_/PTFE on PEVA | 40 | 275  (217 mS) | NCM811/Li | Pressure | 16.5 | RT | 182 | ^[12]^ |
| **Li_6_PS_5_Cl/NBR on h-PI** | **28** | **136  (181 mS)** | **NCM811/ Li-In** | **Pouch** | **17.7** | **RT** | **169** | **This  work** |
|  |  |  |  |  |  | **60** | **197** |  |

^*^ LCO: LiCoO_2_

NCM*xyz*: LiNi_0.x_Co_0.y_Mn_0.z_O_2_

LTO: Li_4_Ti_5_O_12_

Gr: Graphite

PPTA: Poly(para phenylene terephthalamide)

PI: Polyimide

EVAP: Ethyl vinyl acetate porous

LiPSiS: (Li_2_S)_60_(SiS_2_)_28_(P_2_S_5_)_12_

PTFE: Polytetrafluoroethylene

GPE: Gel polymerized electrolyte

P(PEGMEA): Poly(poly(ethylene glycol) methyl ether acrylate)

PEO: Polyethylene oxide

CTMS: (3-chloropropyl)trimethoxysilane

P(VDF-TrFE): Poly(vinylidene fluoride-co-trifluoroethylene)

PEVA: Poly(ethylene vinyl acetate)

^**^ Some ionic conductance data are only presented in mS unit due to lack of information on the areal dimension

Table S4. Thickness and mass of individual components in ASB cells

| Cell component | Thickness (μm) | Mass (mg) |
| --- | --- | --- |
| NCM cathode (1 mAh cm^-2^) | 39 | 9.5 |
| SE pellet | 729 | 150 |
| SES | 27 | 4.8 |
| Li anode | 40 | 2.8 |
| Al current collector | 10 | 3.6 |
| SS current collector | 10 | 10.1 |
| Total mass (with pellet) | 828 | 176 |
| Total thickness (with SES) | 126 | 30.8 |

Table S5. Estimated energy densities of ASB cells with SE pellet and SES, assuming a Li metal anode

| Parameters | SE pellet | SES |
| --- | --- | --- |
| Discharge capacity (mAh g^-1^) | 208.2 | 198.6 |
| Avg. discharge voltage (V) | 3.21 | 3.21 |
| Estimated discharge voltage assuming Li anode (V) | 3.83 | 3.83 |
| Estimated total energy (mWh) | 5.56 | 5.71 |
| Total mass (assuming Li 40 μm, g) | 0.176 | 0.0308 |
| Total volume (assuming Li 40 μm, cm^3^) | 0.110 | 0.0167 |
| Estimated gravimetric ED (Wh kg^-1^) | 31.6 | 186 |
| Estimated volumetric ED (Wh L^-1^) | 50.6 | 342 |


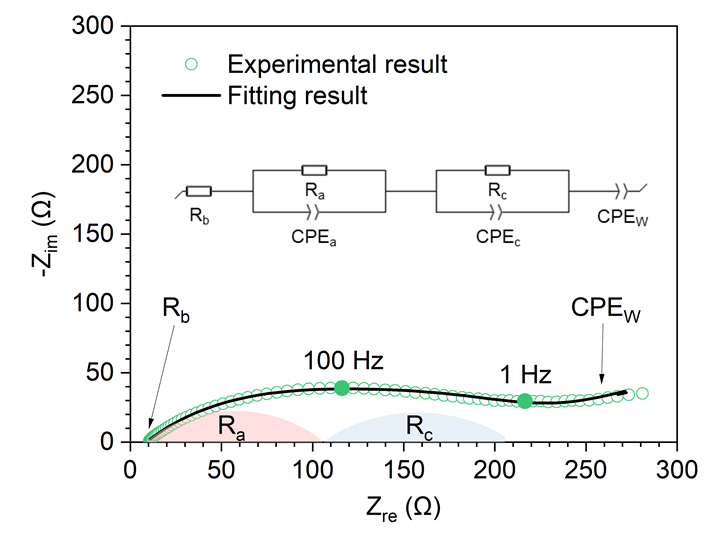


**Figure S9.** EIS result and equivalent circuit model used for fitting. R_b_: bulk resistance, R_a_: anode interfacial resistance, R_c_: cathode interfacial resistance. Constant phase element (CPE) was used to represent an imperfect capacitor. Tail at low frequency could be attributed to Warburg diffusion of Li^+^ within the composite cathode**.**

Table S6. EIS fitting results of SE pellet and SES cells before and after cycling

|  | SE pellet cell | | SES cell | |
| --- | --- | --- | --- | --- |
|  | Before cycle | After cycle | Before cycle | After cycle |
| R_b_(Ω) | 7.82 | 8.55 | 3.81 | 3.78 |
| R_a_(Ω) | 9.60 | 97.2 | 4.17 | 24.4 |
| R_c_(Ω) | 3.61 | 104 | 5.57 | 125 |


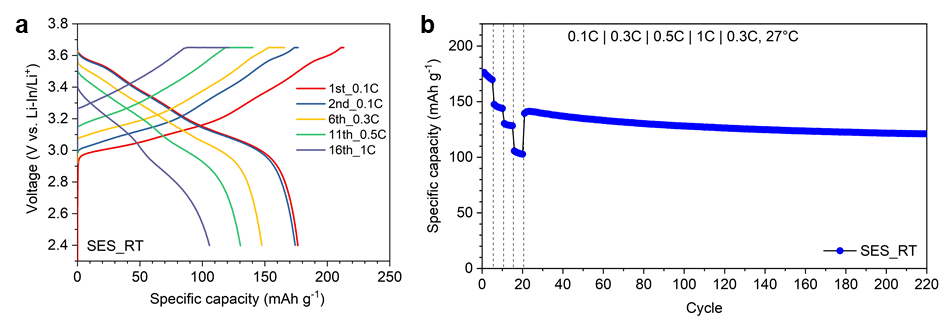


**Figure S10.** (a) Charge-discharge voltage profiles of SES cell measured at 27 °C at various C-rates. (b) Cycle performance of SES cell measured at 27 °C.


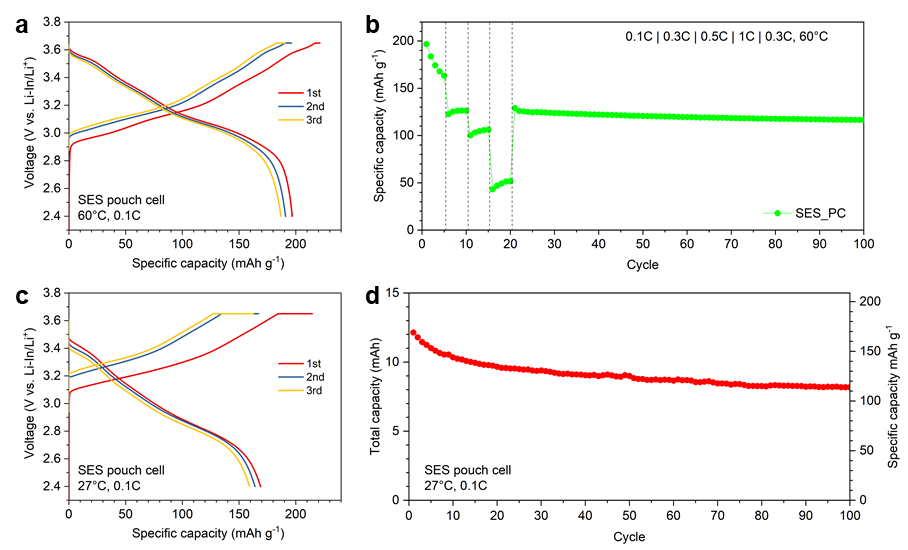


**Figure S11.** (a) Initial charge-discharge voltage profiles and (b) rate capability and cycle performance of SES pouch cell measured at 60 °C. (c) Initial charge-discharge voltage profiles and (d) cycle performance of SES pouch cell measured at 27 °C.

Table S7. Thickness and mass of individual cell components in ASB pouch cell

| Cell component | Dimension (cm^2^) | Thickness (μm) | Mass (mg) |
| --- | --- | --- | --- |
| NCM cathode (3 mAh cm^-2^) | 2 x 2 | 115 | 86.7 |
| SES | 2.4 x 2.4 | 27 | 20.8 |
| Li anode | 2.2 x 2.2 | 40 | 10.3 |
| Al current collector | 2 x 2 | 10 | 10.8 |
| SS current collector | 2.2 x 2.2 | 10 | 30.4 |
| Total |  | 202 | 159 |

Table S8. Estimated energy densities of ASB pouch cell with SES, assuming a Li metal anode

| Parameters | SES pouch cell |
| --- | --- |
| Discharge capacity (mAh g^-1^) | 196.9 |
| Avg. discharge voltage (V) | 3.17 |
| Estimated discharge voltage assuming Li anode (V) | 3.79 |
| Estimated total energy (mWh) | 51.2 |
| Total mass (assuming Li 40 μm, g) | 0.159 |
| Total volume (assuming Li 40 μm, cm^3^) | 0.0898 |
| Estimated gravimetric ED (Wh kg^-1^) | 322 |
| Estimated volumetric ED (Wh L^-1^) | 571 |


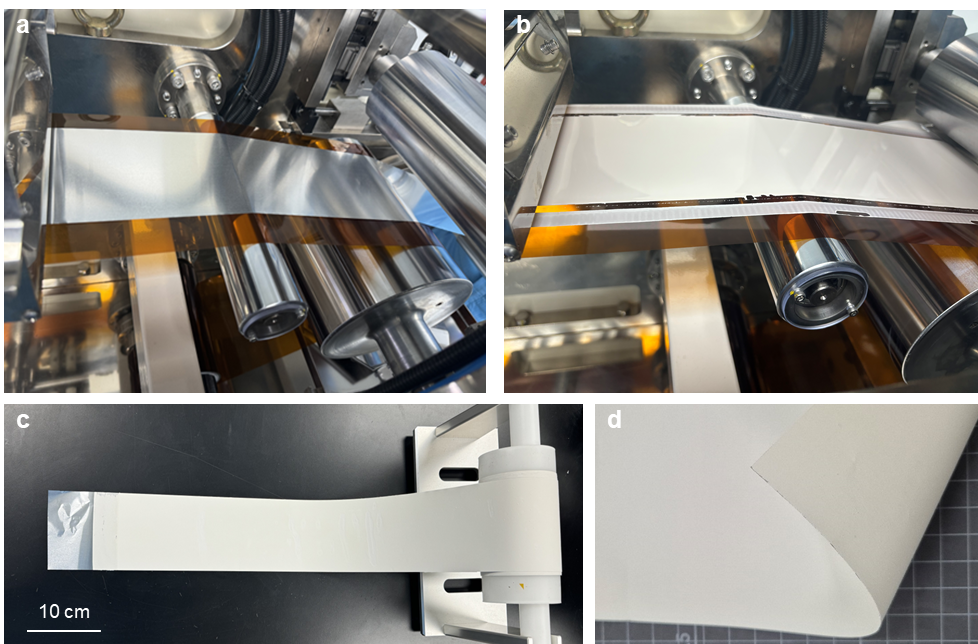


**Figure S12.** Photographs of SES roll-to-roll fabrication: (a) scaffold before coating, (b) immediately after coating, and (c) after drying. (d) Final double-side coated SES produced using a comma coater.

Table S9. Design parameters of the 2D full cell model.

|  |  | D400_G120 | D200_G60 | D100_G30 | D200_G30 |
| --- | --- | --- | --- | --- | --- |
| Solid electrolyte separator (SES) | Total SES thickness (μm) | 30 | | | |
|  | Scaffold thickness (μm) | 8 | | | |
|  | Length in x-axis (μm) | 1040 | | | |
| Cathode | Composition (wt%) | NCM : LPSCl : Super P^®^ Li : NBR = 80 : 18 : 2 : 1 | | | |
|  | Composite density  (g cm^-3^) | 2.87 (Porosity: 15%) | | | |
|  | Composite loading (mg cm^-2^) | 22.13 (Areal capacity: 3 mAh cm^-2^) | | | |

Table S10. Electrochemo-mechanical parameters for Multiphysics simulation.

| Parameter (Unit) | Value |
| --- | --- |
| Ionic conductivity of LPSCl (mS cm^-1^) | 2.0 |
| Electronic conductivity of NCM (S cm^-1^) | 8.5×10^-4^ |
| Electronic conductivity of Super P^®^ Li (S cm^-1^) | 8.7 |
| Diffusion coefficient of LPSCl (m^2^ s^-1^) | 1.0×10^-11^ |
| Transference number of LPSCl | 0.99 |
| Maximum lithium concentration of LPSCl (mol m^-3^) | 42024 |
| Maximum lithium concentration of NCM (mol m^-3^) | 47664 |
| Exchange current density b/w NCM/LPSCl (mA cm^-2^) | 0.1 |
| Exchange current density b/w Li/LPSCl (mA cm^-2^) | 0.1 |
| Specific active surface area of cathode (m^2^ m^-3^) | 1×10^4^ |
| Young’s modulus of scaffold (GPa) | 1 |
| Young’s modulus of LPSCl (GPa) | 22.1 |
| Young’s modulus of NBR (GPa) | 0.01 |
| Poisson’s ratio of scaffold | 0.42 |
| Poisson’s ratio of LPSCl | 0.37 |
| Poisson’s ratio of NBR | 0.41 |

**
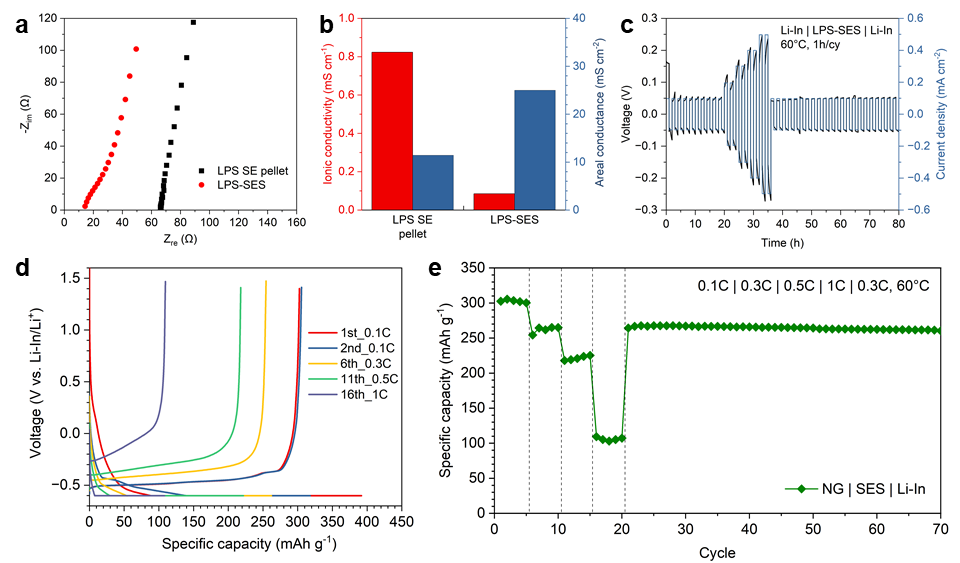
**

**Figure S13.** (a) Nyquist plots of LPS pellet and LPS-SES measured at room temperature. (b) Ionic conductivities and areal conductances derived from the EIS data. (c) CCD test of Li-In|LPS-SES|Li-In symmetric cell at 60 °C at various current densities (0.1 to 0.5 mA cm^-2^). (d) Charge-discharge voltage profiles and (e) cycle performance of NG|SES|Li-In half cell measured at 60 °C.

**References**

[1] Y. J. Nam, S.-J. Cho, D. Y. Oh, J.-M. Lim, S. Y. Kim, J. H. Song, Y.-G. Lee, S.-Y. Lee, Y. S. Jung, *Nano Lett.* **2015**, 15, 3317.

[2] R. Xu, J. Yue, S. Liu, J. Tu, F. Han, P. Liu, C. Wang, *ACS Energy Lett.* **2019**, 4, 1073.

[3] D. H. Kim, Y.-H. Lee, Y. B. Song, H. Kwak, S.-Y. Lee, Y. S. Jung, *ACS Energy Lett.* **2020**, 5, 718.

[4] W. Jiang, L. Yan, X. Zeng, X. Meng, R. Huang, X. Zhu, M. Ling, C. Liang, *ACS Appl. Mater. Interfaces* **2020**, 12, 54876.

[5] T. Yersak, J. R. Salvador, R. D. Schmidt, M. Cai, *International Journal of Applied Glass Science* **2021**, 12, 124.

[6] T. Jiang, P. He, Y. Liang, L.-Z. Fan, *Chem. Eng. J.* **2021**, 421, 129965.

[7] G. L. Zhu, C. Z. Zhao, H. J. Peng, H. Yuan, J. K. Hu, H. X. Nan, Y. Lu, X. Y. Liu, J. Q. Huang, C. He, J. Zhang, Q. Zhang, *Advanced Functional Materials* **2021**, 31, 2101985.

[8] Y. Wang, J. Ju, S. Dong, Y. Yan, F. Jiang, L. Cui, Q. Wang, X. Han, G. Cui, *Advanced Functional Materials* **2021**, 31, 2101523.

[9] H. Liu, P. He, G. Wang, Y. Liang, C. Wang, L.-Z. Fan, *Chem. Eng. J.* **2022**, 430, 132991.

[10] S. Liu, L. Zhou, J. Han, K. Wen, S. Guan, C. Xue, Z. Zhang, B. Xu, Y. Lin, Y. Shen, L. Li, C. W. Nan, *Adv. Energy Mater.* **2022**, 12, 2200660.

[11] S. H. Kang, J. Choi, J. Y. Kim, D. O. Shin, Y.-G. Lee, J. Lee, *ACS Appl. Mater. Interfaces* **2023**, 15, 28064.

[12] D. Li, H. Liu, C. Wang, C. Yan, Q. Zhang, C. W. Nan, L. Z. Fan, *Advanced Functional Materials* **2024**, 34.
